# Traffic safety knowledge gain of ambulance drivers after simulator-based training

**DOI:** 10.1186/s12909-022-03279-w

**Published:** 2022-03-30

**Authors:** Maria J. Prohn, Britta Herbig

**Affiliations:** Institute and Clinic for Occupational, Social and Environmental Medicine, University Hospital, LMU Munich, Ziemssenstr. 5, 80336 Munich, Germany

**Keywords:** Emergency medical service (EMS), Experience, High order training, Subjective and objective knowledge, Test construction, blue lights and sirens

## Abstract

**Background:**

Compared to other road users, ambulance drivers are at a higher accident risk while driving with warning lights and sirens. No standard exists for training or education for emergency medical service employees driving ambulances. Training programs should positively influence knowledge. However, knowledge gain can be influenced by several different factors. This study developed a knowledge test for ambulance drivers to determine influencing factors on knowledge and its gain by simulator-based training.

**Methods:**

Two parallel knowledge test forms with 20 questions each were designed in several steps and tested on up to 174 participants. Questionnaires were used to study associated and influencing factors, such as objective experience, subjective attitudes, personality, motivation and demographic data.

**Results:**

Test construction showed good overall parallelism of the two tests as well as reliability and sensitivity. There was no correlation between subjective and objective knowledge gain, but participants with higher subjective knowledge gain showed a higher variation in objective knowledge. Younger age, higher qualification, higher number of license classes, fewer traffic violations, and more traffic safety trainings were positively associated with knowledge, whereas less yearly driving mileage, more traffic safety trainings, and higher risk sensitivity positively influenced knowledge gain through the training.

**Conclusion:**

Knowledge and its gain through training are very low. Reasons for the lack of predictive power of some variables, such as motivation, personality and attitudes, are discussed. This study presents a new tool for testing knowledge on driving with warning lights and sirens. It shows the need for objective testing and for further research in this special area.

**Supplementary Information:**

The online version contains supplementary material available at 10.1186/s12909-022-03279-w.

## Background

Driving ambulances is a main work task for emergency medical service (EMS) employees to reach the person needing help. In Germany, the national Road Traffic Regulations (Straßenverkehrsordnung, “StVO”) concede special exceptions for ambulances and determine how other road users must behave if a special unit approaches with warning lights and sirens (§35 & 38, StVO). These legal bases of driving with special rights (for a brief explanation of the German regulations relevant for ambulance drivers, see Additional file 1) are comparable to those in numerous other countries. However, driving an ambulance with warning lights and sirens often leads to critical situations and shows a much higher accident rate compared to other road users [1–4]. Additionally, driving with warning lights and sirens does not improve patient outcomes [5, 6]. The higher accident rates are also a major reason for the high injury and fatality rates of EMS personnel [2, 7]. Accidents occur most often at junctions, usually at a red light, and during overtaking situations [1–4, 8, 9]. Accident statistics show that high speeds and tailgating are high risk factors for severe traffic accidents both for ambulances and in general [10, 11]. Despite these data, a survey in the US showed that some ambulance drivers believe 75 miles per hour (~ 120 km/h) to be a reasonable speed when responding to emergencies [12].

Training programs might have the potential to positively influence road safety. Two types of trainings can usually be found, namely, low- and high-order trainings [13]. Low-order trainings are skill-based and focus mostly on handling and maneuvering vehicles in critical situations. In contrast, high-order trainings focus on insights into driving behaviors while reflecting on one’s own driving performance to anticipate and avoid critical situations instead of needing to address them [14]. Although driver training has the potential to positively influence knowledge [15–17] or behavior [18–20], changing behavior does not always reduce traffic crashes or crash risk [21, 22].

Essential factors for the transfer of knowledge into behavior in general, that is, mostly investigated outside the field of road safety, are motivation and volition [23]. Meta-analyses show a strong impact of personality traits, knowledge, and organizational variables on motivation and, in turn, motivation on learning outcomes and transfer [24, 25]. Declarative knowledge was related to cognitive ability, motivation, self-efficacy, locus of control, conscientiousness, anxiety, openness to experience, age, sex, and climate [24, 26, 27]. Procedural knowledge, in terms of skill acquisition, was related mostly to support and pretraining self-efficacy [24, 25].

In addition, personality traits in particular, extraversion, conscientiousness and agreeableness have been found to be associated with traffic accident involvement, crash risk or driving behavior [28, 29].

The driver’s experience, especially in dangerous situations, can influence their driving habits. However, findings show that drivers with previous ambulance accidents were more likely to be involved in other accidents and were responsible for most injuries after such collisions [2, 7]. Ambulance drivers have more driving experience than do average drivers in Germany [13]. More experience, however, does not necessarily lead to more knowledge [10, 30]. As all these studies show complex relationships and many influencing factors between knowledge, knowledge transfer and behavior, accurate measurement of knowledge in training evaluations is crucial.

Since no knowledge test for special requirements when driving with warning lights and sirens exists yet, the first research question was: Is it possible to develop a reliable, valid and change-sensitive knowledge test for ambulance drivers?

Because the literature shows a number of factors, such as experience, attitudes, and personality, that explain whether knowledge is acquired, the second research question of the study was: What associated and influencing factors can be identified for knowledge and its gain among ambulance drivers?

These research questions were examined as part of a controlled evaluation of high-order training for ambulance drivers reported elsewhere [13].

## Methods

### Intervention

The German Traffic Safety Association, together with the section fire service and salvage of the German Social Accident Insurance, developed a one-day higher-order simulator-based training [31]. The training contains a combination of theoretical and practical content and focuses on avoiding dangerous traffic situations. Participants and trainers determine typical accident risks of ambulance driving and collect the trainees’ own experiences with traffic accidents or incidents. The training covers the following topics: legal basis of ambulance driving, self-perceptions, motivation, and attitudes of the trainees, driving physics, perception, and information processing. This theoretical part is followed by simulator-based training: while one participant completes one of several 5-min simulator rides, the other participants are asked to observe different driving characteristics, such as speed, use of sirens or critical incidents. This process is repeated until each trainee has driven twice, with the second simulator drive being more difficult (e.g., worse weather, more critical alerting keyword or more distraction by the dispatch center). Based on these simulations, participants work with the trainer to determine driving habits that they can use on their next mission to help avoid dangerous driving situations. All participants of our study joined the one-day simulator-based training and were divided into an intervention group, which received training between knowledge tests, and a control/waiting group, which received training after both knowledge tests (see Procedure).

### Statistical analysis

All analyses were performed using IBM SPSS Statistics 25.

Research question one: To calculate item differences in the knowledge test construction, we used chi-square tests, item difficulties and discriminatory power of all items and tested parallelism using the Mann–Whitney test. Reliability and validity were tested with Pearson correlation. For test sensitivity analyses of variance with repeated measures were used, with time as the within-subject factor and study group as the between-subject factor, controlling for age and sex.

Research question two: Descriptive statistics and differences between study groups were calculated using chi-square tests and analyses of variance. To test for factors influencing knowledge and knowledge gain, two hierarchical linear regressions were used. To also capture trends, significance levels up to 0.10 are marked and reported; to account for multiple testing, the Bonferroni adjustment level is also reported where relevant.

Analyses were performed either on the full sample or only the intervention group or the control/waiting group depending on the possible training influence. The full study sample was used to test sensitiveness and influencing factors on knowledge. Tests for validity and knowledge gain were performed with the intervention group. To test reliability, only the control/waiting group was used.

### Knowledge test

#### Development of the knowledge test

The knowledge test was designed to test for learning effects of ambulance drivers in relation to traffic safety. The construction was carried out in different steps.

The first step was to formulate questions concerning traffic safety, especially for driving with warning lights and sirens. Training content, which included a large amount of relevant knowledge, was used for test construction. Experts (i.e., traffic psychologists and emergency medical service employees) formulated all questions and possible answers to construct a highly content valid test. To test for item difficulty and comprehensibility, 10 participants with experience in the German EMS (mean age: 33.1 years, range: 24 to 56 years) answered all 26 developed items. For two items, no one provided the correct answer; for all other questions, item difficulties were between 10.0 and 91.0 (mean: 43.0; for explanations, see footnotes to Table 4).

In the next step, experts changed some of the wording to increase comprehensibility. To ensure that repeated testing to measure knowledge gains did not show gains based on mere recognition, parallel forms of testing were developed: Existing items were checked for already existing parallelism, or parallel items were developed. Some questions were used in both test forms but with different answer choices. Some parallel items were designed with different orders of answer choices and slightly different settings of tasks. Answer formats for most items were multiple choices with one correct answer. In addition, some questions had an order format where four answers had to be sorted into the correct order or had multiple correct answer responses. One free response question was used in both parallel test versions. The results were two parallel test forms with 20 questions each.

In a second evaluation step, 10 further participants without experiences in the EMS answered these parallel test forms to control for parallelism and especially the comprehensibility of questions to validate items [32]. Two items were reformulated to obtain a better understanding.

The item order of parallel test items was different for both tests except the situational judgment question, which was the final question in both test versions. The final versions of the tests are presented in more detail in the next section and can be found in the appendix (for German versions, see Additional file 2 and Additional file 3; for the English ad hoc translation, see Additional file 4).

#### Final knowledge test

Both parallel test forms of the final knowledge test used for pre- and posttests contained 19 multiple choice questions with content such as accident background, legal bases, characteristics of ambulance driving, driving physics, perception or attitude toward traffic safety and one additional free response question. Table 1 gives an overview of all parallel items with their response format and scoring. Respondents were not penalized for incorrect answers.

**Table 1:** Item formats and rewards of correct answers of the final knowledge test

Response format	Items	correct responses	scoring (points)
Multiple choice	A1/B12, A3/B18, A4/B1, A5/B14, A6/B5, A7/B15, A8/B11, A10/B4, A11/B8, A12/B7, A13/B19, A14/B2, A15/B10, A16/B16, A18/B3, A19/B9	1 of 5	1 each
Sorting of 4 responses	A2/B17	right order	1 if all correct
Multiple correct answers	A9/B6 A17/B13	2 of 4 5 of 8	0.5 each 0.2 each
Free responses	A20/B20	5	1 each category^a^
*Maximum possible points to reach*			*26.5*

^a^see also coding system below and Additional file 5 for scoring of free responses

Questions 1 to 19 of the knowledge test capture mostly declarative knowledge on traffic safety, whereas with the final open response format question, we aim to explore procedural knowledge, that is, know-how of safe ambulance driving. As this knowledge determines the transfer of knowledge into action, this question was weighted higher, and the responses were coded very carefully. The 20th question aimed to place participants in a typical - or for some drivers, stressful - situation in which they think of possible actions to reach the operation site safely. They could list up to five free concrete responses they believed to have the highest impact on safe driving to an emergency site.

To rate free responses, training content was used and all responses were sorted into seven categories important for ambulance driving or into one additional category for general activities that led to safer driving or fewer injuries. The categories are as follows:

1. minimize distraction or set priorities (1 point)
2. route planning (1 point)
3. usage of right of way and warning light and sirens (1 point)
4. speed and driving physics (1 point)
5. scope of action for other road users (1 point)
6. decision behavior (1 point)
7. information processing and personal requirements (1 point)
8. general activities (independent of blue light driving) (0.5 points)

After defining the categories, we operationalized them with numerous examples. Additionally, examples of answers that were too general were given that would not be scored (e.g., “stay calm”, “be attentive” or “not be stressed”; see Additional file 5). To test the coding system, 19 participants were randomly selected from the sample and assessed by two different raters. A total of 85.2% of the 304 ratings were consistent, and the intraclass correlation coefficient (two-way mixed, type agreement) was ICC_(3,2)_ = 0.753. Discrepancies were discussed, the coding system was adapted, and new examples were added. In the next step, another 20 participants were randomly selected and assessed by the same two raters. The consistency was 93.8% with a very good ICC_(3,2)_ = 0.909.

After the final coding system was determined, one rater assessed the free responses of all participants and consulted the second rater only when undefined answers were given. Each named category was counted once per test. If one response included more than one category (for example, “I tell my co driver [1-minimize distraction] to search for an alternative route [2-route planning].”), participants were awarded for each named category.

In general, the knowledge test was designed as a power test, so each participant had as much time as she or he needed to complete it.

### Procedure

The main evaluation study [13] was a controlled intervention study with an intervention group (Group 1) and control/waiting group (Group 2). The intervention group received training between pre- and posttests, and the control/waiting group received training after both tests. Posttests were conducted between 1 and 2 months after training for the intervention group and between 1 and 2 months after the first test but directly before training for the control/waiting group. Since this study was a field study, assignment to study groups was not random but corresponded to opportunities for data collection and the training date.

The participants had to complete the tests without help from others under the supervision of the experimenter or the trainer. Knowledge tests took 15 min on average.

Parallel knowledge test versions A and B were equally distributed to participants using study group, age, organization, and gender as stratification variables. Table 2 shows the distribution of these variables. No age difference was found between the test version orders in either study group (intervention group: F_(1,94)_ = 0.67, *p* = 0.414; control group: F_(1,74)_ = 0.03, *p* = 0.865). The chi-square test did not show a difference between test distributions concerning the gender of participants (p_intervention_ = 0.639; p_control_ = 0.782) or rescue organizations (p_intervention_ = 0.893; p_control_ = 0.833) in either study group (see Table 2).

**Table 2:** Distributions of age, gender and organization in both test orders for both study groups

Test order		Intervention group		control/waiting group	
		A then B	B then A	A then B	B then A
Age	Mean (years)	35.6	33.9	31.2	31.5
Gender	Female	11	10	9	8
	Male	34	39	28	29
Rescue Organization	organization 1	6	4	3	4
	organization 2	22	28	22	24
	organization 3	4	4	1	2
	organization 4	7	8	5	3
	organization 5	6	5	6	4
**Sum**		**45**	**49**	**37**	**37**

### Participants and recruitment

Participants were rescue workers, emergency medical technicians (EMT), paramedics, and emergency paramedics (for an overview of the qualifications, see Additional file 5) of rural and urban areas of Saxony and southern Bavaria, Germany. All rescue services sited in the areas of data collection were informed and invited to participate in the study. Twenty-six out of 52 operators participated with their employees, including at least one participant from all rescue services sited in Germany (German Red Cross, Federation of Samaritan Workers, Order of Malta Volunteers, St John Ambulance and private rescue services). The inclusion criterion for voluntary participants was a driving time of 40 h per month on vehicles usually driving with warning lights and sirens. Sample size estimation based on analysis of variance (alpha-error of 10%; power of 0.80) with small to medium effect sizes to be detected led to 200 participants [33]. As long as the sample size was not reached, every eligible participant could join the study. All participants signed a written informed consent form indicating the data privacy policy, all planned tests, contact information and withdrawal rights. The Ethics Committee at the Faculty of Medicine of Ludwig-Maximilians-University Munich approved the study and its processes (ID: 206–14).

Overall, 217 persons showed interest in participating in the main study, of which 27 (12.4%) dropped out before start of study and another seven (3.2%) before any data were collected. Of the 183 participants who started measurements, 174 participants (95.1%) completed the first knowledge test. Due to not completing the second knowledge test, six participants (3.4%) were dropped from further analyses, which had no significant influence on demographic data. For knowledge gain, the measures of 94 of the 174 participants (intervention group) who received training could be used for analyses. Table 3 gives an overview of the demographic data of both groups.

**Table 3:** Demographic data for all participants with at least one knowledge test and for intervention group

	Participants for tests of knowledge, total sample	Participants for tests of knowledge gain, intervention group
Sample Size	*N* = 174	*n* = 94
age	33.3 ± 9.5 years [20, 65]	34.8 ± 10.1 years [20, 65]
gender	21.8% female 78.2% male	22.3% female 77.7% male
qualification	2.9% rescue workers 23.6% EMT 72.4% paramedic 1.1% emergency paramedics	2.1% rescue workers 21.3% EMT 75.5% paramedic 1.1% emergency paramedics
working experience	129.3 ± 103.3 months [4, 456]	149.4 ± 111.8 months [10, 456]
driving experience	14.7 ± 9.0 years [1, 44]	16.3 ± 9.6 years [2, 44]

### Impact factors for knowledge and knowledge gain

#### Objective experience

Drivers’ experiences can be divided into traffic and EMS experiences. Experience in EMS was measured with qualification (0 “EMT or lower” and 1 “paramedic or higher”), number of months working in EMS and employment type (0 “part-time or voluntary” or 1 “full-time”). Traffic experience was measured using years of license possession (in years), number of licenses (0 “European Class B and C1” and 1 “further licenses, such as A, C or E”), yearly driving mileage (in km), traffic violations (0 “no past violation” or 1 “past violations”) and past traffic safety trainings (0 “no past training” and 1 “at least one traffic safety training”).

#### Subjective attitudes

Subjective attitudes toward traffic safety were measured using self-assessment of driving skills and risk sensitivity. To measure self-reported driving skills, three items of the Driving Skill Questionnaire [34] were used in their original form (comparison to average drivers) as well as using ambulance drivers as a comparison group (e.g., “Compared to other ambulance drivers, how safe do you think your driving is?”) [35]. All items were scored on an 11-point Likert scale ranging from 0 “much worse/much lower than average” to 11 “much higher/much better than average”. Skillful and safe driving were combined into driving competence (Cronbach’s alpha of 0.85 for average drivers, Cronbach’s alpha of 0.89 for ambulance drivers), whereas accident likelihood remained as a single item.

To measure risk sensitivity, items from the Risk Perception Questionnaire [36] and the driving offences questionnaire [37] were used. Overall, 30 items describing driving situations (e.g., “Drive the wrong way up a one-way-street”) were rated on a 5-point Likert scale ranging from “not at all risky” (1) to “very risky” (5). After assessing stability and reliability, four subscales with 27 items were derived. Subscales were risk sensitivity for a) serious rule violations (nine items, Cronbach’s alpha = 0.62; e.g., “Driving after drinking two beer cans”), b) moderate rule violations (six items, Cronbach’s alpha of 0.73 (e.g., “Drive the wrong way up a one-way-street”), and c) light rule violations (six items, Cronbach’s alpha of 0.71; e.g., “Driving after a sleepless night”). Another six items referred to d) risk sensitivity in regular driving situations with a Cronbach’s alpha of 0.69 (e.g., “Driving a steep descent in a high gear”).

#### Personality

To measure personality-related factors, we used some items from a validated German scale for traffic-related personality [38]. All items were evaluated on a 4-point Likert scale ranging from “not true” (1) to “true” (4). After evaluating internal consistency, new subscales were developed. Seven items measured traffic-related extraversion (Cronbach’s alpha = 0.80; e.g., “when the street is free of traffic, I strongly accelerate”), and another four concerned sensation seeking (Cronbach’s alpha = 0.60; e.g., “the more challenging the driving situation is, the more fun I have”). Conscientiousness was measured with three items (Cronbach’s alpha = 0.58; e.g., “when I don’t have right of way, I double check before I start driving”). Five items each measured disagreeableness (Cronbach’s alpha = 0.74; e.g., “I once drove a car even though I actually was too tired to drive”) and reactance (Cronbach’s alpha = 0.53; e.g., “I drive as I want to; nobody is allowed to instruct me how to drive”).

#### Training motivation and subjective knowledge gain

Motivation is an important impact factor in learning. To measure this control variable, we used one item of the evaluation measured directly after training (“I am motivated to use the knowledge I learned in actual ambulance driving”) on a 5-point Likert scale (1=“not at all” to 5 = “completely”). This scale also measured participants’ subjective knowledge gain in general (“I learned a lot in this training”), and specific knowledge gain on traffic safety (“I found alternatives to drive more safely”). The three items together form a scale for subjective knowledge gain (Cronbach’s alpha = 0.88). Training motivation and subjective knowledge gain could only be controlled for the intervention group since the control/waiting group did not receive training between both knowledge tests.

#### Demographic data

The demographic data used to control for influencing factors for knowledge and learning were age in years and gender (1 “female” and 2 “male”).

## Results

### Test construction

#### Item difficulty and discriminatory power

The final knowledge test forms were analyzed with regard to item difficulty and discriminatory power of all items as well as internal consistency of the tests. Since training contents should have an influence on the question responses, we used the second test of the intervention group (*N* = 94) to test for parallelism. Item difficulties and discriminatory power for all items of both test versions are described in Table 4.

**Table 4:** Item difficulties and discriminatory power of parallel items of both test versions after training

A				B			
Item	Difficulty	SD	discriminatory power	Item	Difficulty	SD	discriminatory power
1	12.50	0.33	0.104	12	36.96	0.49	0.145
2^b^	10.42	0.31	0.154	17^b^	17.39	0.38	0.223
3	89.58	0.31	0.054	18	21.74	0.42	0.242
4	47.92	0.50	0.163	1	52.17	0.51	0.183
5	33.33	0.48	0.001	14	65.22	0.48	0.225
6	37.50	0.49	−0.145	5	30.43	0.47	0.389
7	85.42	0.36	0.070	15	80.43	0.40	0.176
8	33.33	0.48	0.026	11	39.13	0.49	0.051
9^a^	89.58	0.25	0.019	6^a^	77.17	0.33	0.066
10	50.00	0.51	0.052	4	39.13	0.49	0.071
11	56.25	0.50	0.001	8	45.65	0.50	−0.028
12	22.92	0.42	0.161	7	10.87	0.31	0.165
13	33.33	0.48	0.207	19	17.39	0.38	0.331
14	37.50	0.49	−0.110	2	34.78	0.48	−0.055
15	18.75	0.39	0.003	10	21.74	0.42	0.198
16	22.92	0.42	−0.117	16	30.43	0.47	0.023
17^a^	70.00	0.28	0.053	13^a^	79.57	0.19	0.115
18	45.83	0.50	0.008	3	43.48	0.50	0.028
19	41.67	0.50	0.143	9	26.09	0.44	0.253

Note: *n* = 94 participants (intervention group); Number of test item in A matches the number of test B in the same line

^a^multiple correct answer options

^b^sort answers in right order; rest- five response options, item difficulty is the proportion of people answering the item correctly, that is, the higher the value the easier is the question; item difficulty usually has no unit but describes percentages

Discriminatory power is low for all items as well as internal consistency of all multiple choice items (test A α = 0.159; test B α = 0.461). These criteria show that the knowledge test itself does not measure one knowledge part but different contents of knowledge.

More than half the items show medium item difficulties (between 30 and 70) and therefore good separation efficiency. Higher and lower item difficulties might help to differentiate between participants with very high or very low knowledge. Differences between both test versions exist for three items.

In one item (Test A question 1, Test B question 12) the differences (*p* = 0.006) are not explicable since there is actually no difference in the question besides another order of the response options, therefore they will remain in further calculations. The two other items concern legalities. Test A question 3 with its parallel item Test B question 18 include the different contents of the paragraphs 35 and 38 StVO which are often mixed-up by emergency personnel and it seems that the training cannot change that fact. Version A has more correct responses (*p* < 0.001). However, this question is quite important to measure legal knowledge and therefore remained in the analyses. The other item (Test A question 5, Test B question 14) deals with behavior after having an accident on the way to an emergency. Here, version B shows more correct responses (*p* = 0.002). This item is also an important question that all people driving an ambulance should be able to answer correctly and remains in the final analyses.

#### Reliability and validity

Reliability was tested via test-retest reliability correlating the different test versions in the control−/waiting-group (*n* = 74) that had no training between both test versions. Correlation between both test versions is *r* = 0.358 (*p* = 0.002) and therefore test-retest reliability for the complete test is small but given.

**Fig. 1:**
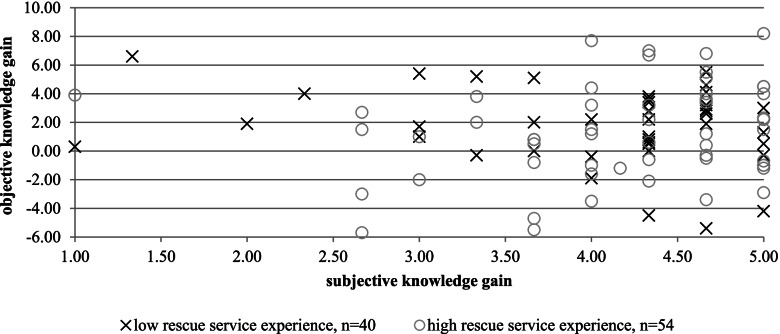
Relation between subjective knowledge gain directly after training and objective knowledge gain (*n* = 94, intervention group)

There is no correlation between subjective and objective knowledge gain (*r* = 0.010, *p* = 0.923) in the intervention group. However, a scatter plot shows more detailed information on the correlation between subjective and objective knowledge gain (see Fig. 1). Only few participants rate themselves as having a low subjective knowledge gain. The higher the subjective knowledge gain the higher is the variation of the objective knowledge gain. Participants with high EMS experience tend to report higher knowledge gain than participants with lower experience. Overall, participants overestimate their knowledge gain.

#### Sensitiveness

**Fig. 2:**
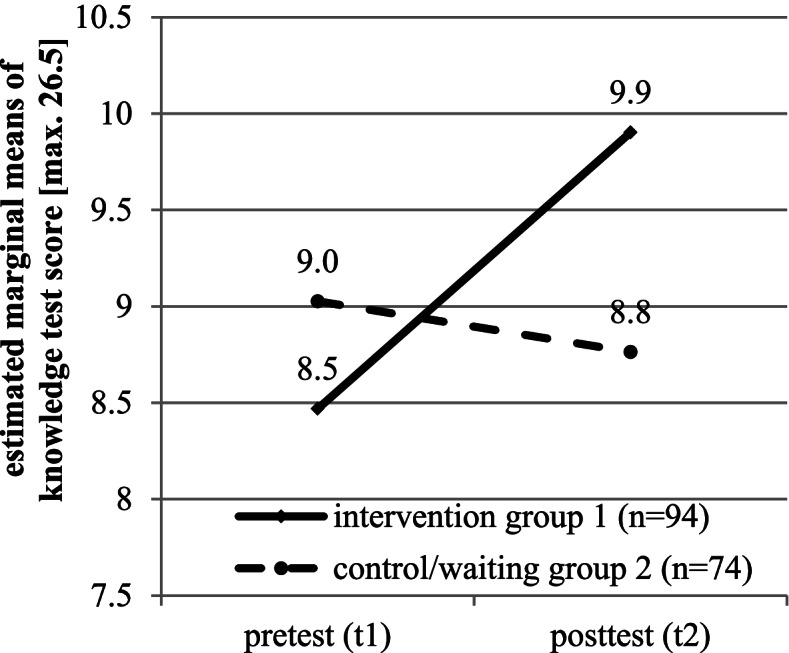
Influence of training on knowledge test results to measure sensitiveness

To measure sensitiveness, we analyzed if a training that should have an effect on learning in the tested area also led to different knowledge test results. Therefore, we compared both study groups with each other at both test times. Both test versions are used in the pretest as well as in the posttest. Since test versions A and B are comparable divided in both groups, all items are used for analyses. Repeated measures analysis of variance controlled for age and sex shows an interaction effect between training group and measuring time (F_(1,166)_ = 12.09, *p* = 0.001, η_p_^2^ = 0.069, Fig. 2). That is, the knowledge test is sensitive.

### Factors influencing knowledge

We analyzed which variables were associated with knowledge before training using a hierarchical regression analysis. In a stepwise procedure demographic data, followed by work and driving experience, attitudes, personality and motivational factors were entered. Table 5 presents the results.

**Table 5:** Factors influencing knowledge

*n = 147*	B	SE	ß	B	SE	ß	B	SE	ß	B	SE	ß	B	SE	ß	B	SE	ß
Step 1: demographic data																		
• age (years)	−0.06	0.02	−0.209*^ǂ^	−0.07	0.04	−0.269^+^	− 0.04	0.10	− 0.155	−0.02	0.10	−0.076	− 0.01	0.10	− 0.035	−0.04	0.11	−0.141
• gender	0.53	0.49	0.090	0.58	0.50	0.099	0.77	0.51	0.131	0.60	0.52	0.103	0.60	0.52	0.101	0.59	0.53	0.100
Step 2: work experience																		
• qualification				0.94	0.52	0.163^+^	1.09	0.51	0.189*	1.16	0.52	0.200*	1.00	0.55	0.172^+^	0.78	0.57	0.134
• EMS duration (months)				0.00	0.00	0.069	0.00	0.00	0.026	0.00	0.00	0.079	0.00	0.00	0.025	0.00	0.00	0.047
• employment type				0.13	0.68	0.018	0.57	0.68	0.076	0.56	0.70	0.075	0.47	0.71	0.063	0.21	0.71	0.029
Step 3: driving experience																		
• license possession (years)							−0.01	0.10	−0.046	− 0.03	0.11	− 0.110	− 0.02	0.11	− 0.074	− 0.00	0.11	− 0.010
• number of licenses							1.13	0.63	0.157^+^	1.22	0.65	0.170^+^	1.21	0.67	0.169^+^	1.12	0.67	0.157^+^
• yearly driving mileage (in km)							−0.00	0.00	−0.136	− 0.00	0.00	− 0.106	− 0.00	0.00	− 0.099	− 0.00	0.00	− 0.089
• traffic violations							−0.96	0.46	−0.192*	− 0.92	0.47	− 0.183*	− 0.92	0.48	− 0.148^+^	− 0.88	0.48	− 0.174^+^
• traffic safety trainings							1.06	0.45	0.199*^ǂ^	1.02	0.46	0.191*^ǂ^	0.84	0.47	0.158^+^	0.91	0.47	0.170^+^
Step 4: subjective attitude																		
• driving competence										−0.16	0.16	− 0.085	− 0.16	0.16	− 0.088	− 0.16	0.16	− 0.088
• accident likelihood										−0.01	0.12	− 0.006	0.02	0.12	0.017	0.00	0.12	0.001
• RS serious RV										0.91	0.88	0.119	1.17	0.90	0.153	0.99	0.90	0.131
• RS moderate RV										−0.84	0.56	−0.186	− 0.67	0.58	− 0.119	− 0.66	0.60	− 0.147
• RS light RV										−0.16	0.64	−0.035	− 0.37	0.67	− 0.082	−0.51	0.67	−0.115
• RS regular driving situations										−0.01	0.53	− 0.002	− 0.09	0.54	− 0.021	− 0.08	0.55	− 0.018
Step 5: traffic related personality																		
• extraversion													0.96	0.67	0.151	0.91	0.67	0.144
• sensation seeking													0.12	0.50	0.022	0.38	0.51	0.069
• conscientiousness													−0.42	0.38	−0.103	− 0.42	0.38	−0.102
• disagreeableness													−0.34	0.50	−0.068	− 0.36	0.50	− 0.072
• reactance													−0.73	0.62	−0.123	− 0.80	0.64	− 0.134
Step 6: Motivation																		
• motivation to use learned																0.61	0.39	0.208
• overall subjective knowledge gain																−0.16	0.38	−0.061
• safety alternatives																0.12	0.38	0.045
***R***^***2***^			.043*			.075*			.165**^ǂ^			.195*^ǂ^			.223*			.254*
***ΔR***^***2***^			.043*			.032			.090**^ǂ^			.030			.028			.031
***F (df)***			3.220 (2144)			2.288 (5141)			2.689 (10,136)			1.966 (16,130)			1.708 (21,125)			1.733 (24,122)

*n* number of participants, *SD* standard deviation, *R*^2^ explained variance, *ΔR*^2^ change in explained variance, *β* beta coefficient with significance level ^+^ *p* ≤ .10, ^*^p ≤ .05, ^**^p ≤ .01, ^***^p ≤ .001

^ǂ^significant after Bonferroni adjustment (*p* ≤ .025); Variables and coding: RS = risk sensitivity of, RV = rule violation; gender (1 = female, 2 = male), qualification (0 = EMT, 1 = Paramedic), employment type (0 = part-time/voluntary, 1 = full-time), number of licenses (0 = B/C1, 1 = additional A, C and/or E), traffic violations & traffic safety trainings (0 = no, 1 = yes)

For demographic data, regression analysis shows an effect of age on knowledge but not of gender. Younger participants showed higher scores than older participants. As for “work experience”, only qualification was found to have a tendency to influence knowledge (on the 10% significance level), in the form that paramedics had higher knowledge scores than EMT. However, the additional variance explained by the step “work experience” is small and not significant. “Driving experience” has the overall highest and significant explanatory power for knowledge in pretests. Participants that had at least one additional license (10% significance level), no traffic violations and at least one past traffic safety training have higher knowledge test scores compared to participants with license B and C1, past traffic violations and no past traffic safety trainings. However, after Bonferroni adjustment just participation in at least one traffic safety training has a significant effect. The steps “subjective attitudes”, “traffic related personality” and “motivation” do not explain additional variance in knowledge test results.

### Factors influencing knowledge gain

Factors that influenced knowledge gain were measured similarly with posttest as dependent variable and pretest results controlled for in step 2 (see Table 6).

**Table 6:** Factors influencing knowledge gain

*n = 84*	B	SE	ß	B	SE	ß	B	SE	ß	B	SE	ß	B	SE	ß	B	SE	ß	B	SE	ß
Step 1: demographic data																					
• age (years)	−0.06	0.02	− 0.209*ǂ	− 0.02	0.03	−0.063	− 0.07	0.06	− 0.257	− 0.24	0.16	− 0.830	− 0.31	0.19	−1.076	− 0.32	0.20	− 1.097	− 0.36	0.21	− 1.231+
• gender	0.53	0.49	0.090	−0.46	0.72	−0.070	−0.71	0.74	−0.108	0.07	0.76	0.010	0.07	0.78	0.011	0.19	0.82	0.028	0.07	0.85	0.010
Step 2: pretest																					
• previous knowledge				0.39	0.12	0.351**ǂ	0.37	0.12	0.339**ǂ	0.28	0.12	0.258*ǂ	0.28	0.12	0.253*	0.33	0.13	0.301*ǂ	0.32	0.14	0.289*ǂ
Step 3: work experience																					
• qualification							0.18	0.79	.027	0.26	0.76	0.038	0.47	0.77	0.070	0.21	0.82	0.032	0.09	0.86	0.013
• EMS duration (months)							0.01	0.01	0.234	0.00	0.01	0.101	0.01	0.01	0.195	0.00	0.01	0.073	0.00	0.01	0.126
• employment type							−0.95	0.90	− 0.120	− 0.43	0.88	− 0.055	− 0.79	0.94	− 0.100	−0.77	0.96	−0.097	− 0.85	0.99	− 0.107
Step 4: driving experience																					
• license possession (years)										0.21	0.16	0.719	0.27	0.19	0.909	0.30	0.20	1.022	0.32	0.21	1.085
• number of licenses										−0.16	0.93	−0.019	− 0.39	0.95	− 0.048	− 0.37	1.02	− 0.045	− 0.32	1.07	− 0.038
• yearly driving mileage (in km)										−0.00	0.00	− 0.269*ǂ	− 0.00	0.00	− 0.142	− 0.00	0.00	− 0.124	− 0.00	0.00	− 0.105
• traffic violations										−0.74	0.68	− 0.129	− 0.74	0.69	− 0.128	− 0.08	0.75	−0.153	− 0.90	0.08	− 0.156
• traffic safety trainings										1.21	0.65	0.203+	0.98	0.66	0.163	1.15	0.71	0.193	1.19	0.73	0.199
Step 5: subjective attitude																					
• driving competence													−0.15	0.23	−0.075	− 0.17	0.26	−0.084	− 0.15	0.26	− 0.074
• accident likelihood													0.18	0.20	0.111	0.25	0.21	0.156	0.24	0.22	0.148
• RS serious RV													3.85	1.30	0.468**ǂ	3.98	1.33	0.484**ǂ	4.08	1.37	0.497**ǂ
• RS moderate RV													−0.49	0.92	−0.94	0.22	1.03	0.042	−0.10	1.10	−0.019
• RS light RV													−1.40	0.97	−0.280	−1.49	1.05	−0.296	−1.48	1.08	−0.295
• RS regular driving situations													−0.03	0.92	− 0.006	− 0.45	0.97	− 0.085	− 0.26	1.01	− 0.049
Step 6: traffic related personality																					
• extraversion																−0.02	0.95	−0.003	0.04	0.97	0.006
• sensation seeking																0.55	0.76	0.095	0.61	0.81	0.105
• conscientiousness																0.43	0.58	0.093	0.41	0.59	0.089
• disagreeableness																1.41	0.78	0.230+	1.30	0.82	0.212
• reactance																−0.22	0.89	−0.035	−0.15	0.93	−0.024
Step 7: Motivation																					
• motivation to use learned																			−0.12	0.68	−0.041
• overall subjective knowledge gain																			0.23	0.58	0.082
• safety alternatives																			0.25	0.62	0.085
***R***^***2***^			.022			.142**^ǂ^			.171*^ǂ^			.303**^ǂ^			.393**^ǂ^			.437*^ǂ^			.446*^ǂ^
***Δ R***			.022			.119***^ǂ^			.029			.132*			.090			.044			.009
***F (df)***			0.931 (2,81)			4.408 (3,80)			2.642 (6,77)			2.845 (11,72)			2.513 (17,66)			2.148 (22,61)			1.864 (25,58)

*n* number of participants of the intervention group, *SD* standard deviation, *R*^2^ explained variance, *ΔR*^2^ change in explained variance, *β* beta coefficient with significance level ^+^ *p* ≤ .10, ^*^p ≤ .05, ^**^p ≤ .01, ^***^p ≤ .001

^ǂ^significant after Bonferroni adjustment (p ≤ .025); Variables and coding: RS = risk sensitivity of, RV = rule violation; gender (1 = female, 2 = male), qualification (0 = EMT, 1 = Paramedic), employment type (0 = part-time/voluntary, 1 = full-time), number of licenses (0 = B/C1, 1 = additional A, C and/or E), traffic violations & traffic safety trainings (0 = no, 1 = yes)

Analyses show a clear influence of prior knowledge on knowledge gain without effects of gender or age. Concerning the further steps of the hierarchical regression analysis, just “driving experience” has a significant explanatory power. Participants with less yearly driving mileage show a significant higher knowledge gain before and after Bonferroni adjustment. Participants who had at least one traffic safety training show a trend to slightly higher knowledge gain compared to participants without training. The steps “work experience”, “subjective attitudes”, “traffic-related personality” and “motivation” do not explain significant additional variance in knowledge gain. However, some single items show an impact or a trend to influence knowledge gain. For attitudes, a significant effect of risk sensitivity of serious rule violations was found before and after Bonferroni adjustment. The higher the assessment of risk sensitivity of serious rule violation the higher was the knowledge gain. Concerning “traffic related personality”, participants who scored higher on the scale disagreeableness tended to show higher knowledge gains.

## Discussion

This study had two goals: developing a change-sensitive, reliable and valid knowledge test for ambulance drivers and obtaining a better understanding of which variables are associated with knowledge and influence knowledge gain in this population.

We have partially achieved the first goal of the study. We developed a test on relevant knowledge for driving with warning lights and sirens with two parallel test forms so that the test can be used in evaluation studies. However, the results of the psychometric properties of the test are ambiguous, although they mostly indicate, at least in part, a reliable, valid and change-sensitive test.

The test for parallelism showed differences especially in legal bases of driving with warning lights and sirens even after a training that focused on these special legal provisions. This result might be due to an implicit simplification of legal bases that ambulance drivers use. Instead of understanding the difference between both relevant legal paragraphs, they assume that other vehicles must yield if they use warning lights and sirens and that they can disregard traffic regulations.

Although we were able to reliably score participants’ free responses (inter-rater reliability, ICC_(3,2)_ = 0.909), the knowledge test reliability was low. The correlation of the complete test results between both test versions was *r* = 0.358 without any intervention in between. This correlation shows that both test versions do not measure exactly the same knowledge content but participants with higher scores on one test version also score higher on the other. Nevertheless, what fraction of the results was affected by the participants simply guessing remains a question (see below).

The concurrent validity of the test forms, measured with subjective knowledge gain, is very low. Participants often overestimated their knowledge gain. Based on the assumption of the very rigid test development that the knowledge test is valid, there are several other explanations for this phenomenon: most prominently, the effects of (over)confidence and effects of memory.

Confidence in oneself and one’s knowledge (gain) has been predominately researched in judgment and decision-making [39, 40] showing that mostly experts, but depending on mass of information, also lays [41], often develop overconfidence in their judgments during the course of addressing a problem. The same mechanism might underlie the effect that subjectively reported knowledge gain is higher than objectively gained knowledge after learning about the topic for 1 day and that this effect seems to be more pronounced for participants with higher EMS experience.

Regarding memory, it might also be that participants were genuinely convinced that they gained knowledge. The time gap between the knowledge tests could have led participants to forget most of the learned content, as the forgetting curve hypothesizes [42]. However, the mere knowledge of having participated in a positively evaluated training could have been used as meta-knowledge for having learned ‘something’. It is also possible that participants learned how to drive more safely with ambulances but did not remember the training content. That is, they may have switched from declarative to procedural knowledge during the time gap. This explanation would be in line with a deliberate practice approach [43]. Although the training did not use this approach theoretically, its practical approach corresponds well with Ericsson’s considerations, i.e., explicating behavioral routines via simulation and feedback to change them.

To obtain a better understanding of the factors associated with knowledge and knowledge gain, we analyzed a number of factors, such as experience, attitudes, motivation and demographic factors. Only a few variables were associated with knowledge in the pretests, namely, age, traffic violation and traffic safety training. Older participants showed lower test scores on this knowledge test than younger participants. This result diverges from the findings of other studies, where older participants showed higher knowledge concerning traffic safety [44, 45]. The specific declarative knowledge usually taught in driving schools might have already faded for older participants, while experience (procedural knowledge) might have increased to fill this knowledge gap. Traffic violation and traffic safety training are both related to driving experience that added the highest but altogether still low amount of explained variance. Higher knowledge test scores were reached by participants with no traffic violations and at least one traffic safety training. The effect of no traffic violations might be an interrelation in both directions. Knowledge of rules might encourage more conscious avoidance of traffic violations. However, no traffic violations might also indicate higher traffic safety awareness and thus a greater willingness to obey the rules.

Regarding knowledge gain, the strongest, albeit rather small, influencing factor was the pre-test, which is unsurprising. Among the other variables tested only yearly driving mileage and risk sensitivity of serious rule violations tended to influence knowledge gain. Participants who drive more had a lower knowledge gain compared to participants who drive less. They also, rated themselves as having greater expertise and had more often traffic violations. This finding might be due to participants’ overconfidence in their driving abilities, similar to the lack of correlation between subjective and objective knowledge gain. If this assumption is correct, it is difficult to change fixed and possibly erroneous knowledge with one-day training. Concerning subjective attitudes, the risk sensitivity of serious rule violations positively impacts knowledge gain. This finding is noteworthy because nearly all participants thought that serious rule violations were dangerous. Therefore, participants with a very high awareness of traffic safety might be more open to new information on this topic.

As with knowledge in pretest, knowledge gain shows that participants who had at least one traffic safety training might be a bit more likely to benefit from the present training. Although not all training is beneficial [21, 22, 46] repeated trainings might help combat forgetting and underlie important content, an effect also found by other authors [47, 48].

A notable finding is the lack of or low explanatory power of subjective attitudes, traffic-related personality and motivation on knowledge and its gain. Nearly all participants rated themselves as having high driving competence and low accident likelihood but scored low on knowledge tests on topics such as traffic rules, accident backgrounds and driving physics even after training. Traffic-related personality did not explain knowledge (gain), which contradicts other studies [49, 50] but confirms the findings of Dean and colleagues [51]. In particular, the lack of effect of motivation must be investigated more precisely in future work since it is known to be an essential factor for transfer, at least for job training [23–25]. The participants in our study made great efforts to participate in the study, which shows their generally strong motivation for this important topic. Additionally, the gap between subjective and objective knowledge gain needs further research to better explain the underlying mechanisms of, for example, experience, confidence or other variables.

## Limitations

Some limitations must be considered when interpreting the results of the present study. First, even though both tests were not parallel overall, all items remained in the final analyses due to reasons discussed before. Second, participants scored low in the pre- and posttests, which might suggest test difficulty that was too high. Low knowledge gain might be caused by the long timeframe between training and testing, and no short-term effect was measured. Often, tests were performed after a shift (that could have been calm but also stressful) and at different times of the day. All of these factors are inherent problems of field research in a highly dynamic setting such as EMS but are comparable between groups. Third, for all self-report questionnaires, participants may have provided socially desirable answers instead of honest answers. However, all questionnaires emphasized the importance of honest answers. Together with assurance of data privacy, the study design tried to decrease socially desirable answers as much as possible, e.g. participants could answer the subjective questions in the absence of the investigators. Generalizability to all ambulance drivers might also be limited. The study was conducted in Germany and is therefore not directly transferable to other countries. However, in order to increase generalizability, several regions within Germany were investigated and all rescue service organizations active there were included.

## Conclusion

In sum and given that there is no knowledge test for driving with warning lights and sirens yet, we were partially successful in our first research aim to develop a reliable, valid and change sensitive knowledge test for ambulance drivers. As discussed, not all psychometric properties of the developed knowledge test were satisfying, but it remains a topic for further research whether these problems are inherent to testing this type of knowledge or in fact problems of the developed test.

For the second research question on factors associated with knowledge and its gain for ambulance drivers, several noteworthy relations were found and discussed. The researched training led to higher knowledge scores but only to a small degree. There is high potential for further knowledge gain. Differences between subjective and objective knowledge gain must be addressed by future trainings. Knowledge gain does not necessarily change behavior. Therefore, the transfer of knowledge into behavior also needs further research. Additionally, the positive effect of at least one previous traffic safety training on knowledge and by trend on knowledge gain may suggest that it is important to frequently repeat trainings to combat forgetting and underline important content.

## Supplementary Information

**Additional file 1.** Traffic Regulations and Qualifications. Gives an overview of the relevant sections of the German Road Traffic Regulations for driving with warning lights and sirens and of the four non-physician qualifications of the emergency medical services in Germany.

**Additional file 2.** Wissenstest-Version A. Shows final knowledge test version A in its original German version.

**Additional file 3.** Wissenstest-Version B. Shows final knowledge test version B in its original German version.

**Additional file 4.** Knowledge test items version A and B – Ad hoc translation (not used or validated). Shows the parallel test items of both final knowledge test version A and B ad hoc translated into English. The English version is not validated nor was it used for study. The correct answers are highlighted in italics with an additional *.

**Additional file 5.** Categories of free responses. Shows all categories for free responses of the knowledge test with explanation and examples for correct and incorrect answers.

## Data Availability

The datasets used and/or analyzed during the current study are available from the corresponding author on reasonable request.

## Abbreviations

EMS: Emergency medical service
EMT: Emergency medical technician
