# Supplementary material for: Traffic safety knowledge gain of ambulance drivers after simulator-based training
Source: BMC Med Educ. 2022 Mar 30;22:216. doi: 10.1186/s12909-022-03279-w (PMC8969364; doi:10.1186/s12909-022-03279-w)
Supplement: Supplementary file 1 — Additional file 1. Traffic Regulations and Qualifications. Gives an overview of the relevant sections of the German Road Traffic Regulations for driving with warning lights and sirens and of the four non-physician qualifications of the emergency medical services in Germany. [file 12909_2022_3279_MOESM1_ESM.pdf]

Additional file 1. Traffic Regulations and Qualifications

Summary of the most important paragraphs of the German Road Traffic Regulations for ambulance drivers

Section 1 – Basic Rules

This section includes the need for constant attention and mutual consideration in the traffic context. Nobody should be harmed, endangered, or hindered if avoidable. It is the basis for the entire Road Traffic Regulations and should always be followed. This applies also for ambulance drivers no matter if they are on a mission or not.

Section 35 – special rights

This section regulates exceptions from the National Traffic Regulations. For ambulance drivers this means that they are exempt from the regulations when utmost urgency is required to save human lives or to avert serious damage to health. However, all exemptions can just be exercised with regard to public safety and order. Exemptions could be for example: driving faster than allowed, driving through a red light, overtake where it is not allowed or driving the wrong way down a one-way street; all exemptions apply only if other road users are not endangered as a result.

The section only exempts ambulance drivers from the regulations of the National Traffic Regulations but not from other legal provisions like the regulations of the Road Traffic Licensing Authority (StVZO) or the Road Traffic Act (STVG). This means, for example, that an ambulance driver on a mission is not allowed to drive without the required driver's license or under the influence of alcohol or drugs.

The decision to use special rights and implementation responsibility of the special rights lies with the driver. She/he alone decides on how to violate traffic regulations and she/he has to deal with possible consequences if a crash happens. Important to note is that this section does not mention warning lights and sirens. The exemption of the regulations is independent of them and is regulated in paragraph 38.

Section 38 – Flashing blue lights and flashing amber lights

This section regulates when flashing warning lights with or without a siren are supposed to be used and what this means for other road users. Warning lights and sirens combined are only allowed in order to safe human life, ward of serious damage to health or danger to public safety and order, to pursue persons on the run or to protect objects of great value. For all other road users this mandates that they have to make way immediately.

Blue warning lights alone do not order other road users to do something specific. They only warn of accidents, incidents, emergency runs, escorting vehicles or driving in close formation. For ambulance drivers usually just the first two reasons mentioned in § 38 are relevant for using warning lights and sirens. These are the same reasons for which they are exempt from traffic rules (see section 35). Only if both, warnings lights and sirens, are used together other road users are obliged to make way. Using only warning lights alone might show that they are on an emergency run. This will often lead to other road users making way; however, they do not have to.

Additional file 1. Traffic Regulations and Qualifications

Non-physician qualifications of emergency medical services (EMS) in Germany

Emergency medical services (including education, training, regulations or permissions) differ between German federal states. This amendment just gives a short overview on the different non-physician qualifications existing in Germany.

qualification	German term	Short description	Training duration	Training parts	Typical role in EMS
Emergency Paramedic (EP)	Notfall-sanitäter	highest EMS qualification implemented in 2014 (officially recognized occupation requiring formal training)	3 years	○ 1920h vocational school<br>○ 720h different hospital departments<br>○ 1960h emergency medical rescue station acknowledged for education<br>○ Final exams (written, verbal and practical)	○ co-driver on RTV<br>○ driver on DV<br>○ driver on RTV if another EP is co-driver<br>○ can act as co-driver on PTA
Paramedic (P)	Rettungs-assistent	until 2013 highest EMS qualification (officially recognized occupation requiring formal training)	2 years	○ 1200h vocational school (incl. hospital departments)<br>○ 1600h emergency medical rescue station acknowledged for education<br>○ Final exams (practical, theoretical, verbal and written)<br>○ can acquire EP with official supplementary test (dependent of professional experience with or without further training)	○ co-driver on RTV<br>○ driver on DV<br>○ driver on RTV if another P or EP is co-driver<br>○ can act as co-driver on PTA
Emergency Medical Technician (EMT)	Rettungs-sanitäter	until 1989 highest EMS qualification, now usually assistant of P or EP (not officially recognized occupation requiring formal training)	520 hours	○ 160h basic course<br>○ 160h different hospital departments<br>○ 160h emergency medical rescue station acknowledged for education<br>○ 40h advanced course<br>○ Final exams (written, verbal and practical)	○ driver on RTV<br>○ driver on DV (in some states)<br>○ co-driver on PTA<br>○ driver on PTA if another EMT or P/EP is co-driver
Rescue Worker (RW)	Rettungs-(dienst)-helfer	differs between federal states	160 to 320 hours	○ 80/160h basic course<br>○ 0/80/160/180h practical training (hospital and/or medical rescue station)<br>○ Final exam (written and practical)	○ driver on PTA<br>○ driver on RTV (in some states)

Note: RTV-Rescue transport vehicle (“RTW”) – usually driving with warning lights and sirens; DV-Doctor’s vehicle (“NEF”) – usually driving with warning lights and sirens; PTA-Patient transport ambulance (“KTW”) – usually not driving with warning lights and sirens; Co-Driver usually is the medical responsible person on each of the vehicles, 2 people are usually on them
