# Supplementary material for: Traffic safety knowledge gain of ambulance drivers after simulator-based training
Source: BMC Med Educ. 2022 Mar 30;22:216. doi: 10.1186/s12909-022-03279-w (PMC8969364; doi:10.1186/s12909-022-03279-w)
Supplement: Supplementary file 2 — Additional file 2. Wissenstest-Version A. Shows final knowledge test version A in its original German version. [file 12909_2022_3279_MOESM2_ESM.pdf]

Additional file 2. Wissenstest-Version A

1. An welchen Orten finden die meisten Unfälle bei Fahrten unter Sonder- und Wegerecht statt? Bitte wählen Sie die korrekte Reihenfolge für absteigende Unfallhäufigkeit aus beginnend mit dem Ort mit der größten Unfallhäufigkeit! *[nur eine Antwort]*

   ☐ Kreuzung, Gerade, Kurve, Einmündung, Ausfahrt

   ☐ Gerade, Kreuzung, Ausfahrt, Kurve, Einmündung

   ☐ Kreuzung, Gerade, Einmündung, Ausfahrt, Kurve

   ☐ Kreuzung, Kurve, Gerade, Einmündung, Ausfahrt

   ☐ Gerade, Kurve, Kreuzung, Ausfahrt, Einmündung

2. Ordnen Sie die Quellen für Unfallursachen in die richtige Reihenfolge, indem Sie die entsprechende Zahl von 1-4 davor schreiben (1=häufigste Ursache, 4=seltenste Ursache).

   ______ Fahrzeugbeherrschung

   ______ Fahreinstellung und -motivation

   ______ Verkehrsregelkenntnis

   ______ Wahrnehmung und Gefahrenerkennung

3. Wie müssen Fahrten unter „Wegerechten“ (§38 der StVO) anderen Verkehrsteilnehmern deutlich gemacht werden? *[nur eine Antwort]*

   ☐ gar nicht

   ☐ durch blaues Blinklicht

   ☐ durch blaues Blinklicht und das Martinshorn

   ☐ durch das Martinshorn

   ☐ durch blaues Blinklicht und Warnblinklicht

4. Bei wie viel Prozent der Unfälle mit Blaulichtbeteiligung sind die Einsatzfahrer/-innen Hauptverursacher? *[nur eine Antwort]*

   ☐ ca. 30% ☐ ca. 37% ☐ ca. 49% ☐ ca. 65% ☐ ca. 78%

5. Stellen Sie sich folgende Situation vor: Sie fahren einen RTW auf dem Weg zum Einsatzort mit dem Stichwort „Atemprobleme bei Kind“. In einer engeren Straße steht ein PKW auf der rechten Seite etwas weiter in der Straße als die restlichen Fahrzeuge. Sie fahren versehentlich den linken Spiegel des PKW ab. Sie unterbrechen die Fahrt und verschaffen sich einen Überblick über die Situation. Welche der folgenden Vorgehensweisen ist im Weiteren die beste? *[nur eine Antwort]*

   ☐ Nachdem nur der Spiegel beschädigt wurde, entscheiden Sie sich, weiter zum Einsatz zu fahren, da ein Menschenleben wichtiger als ein Autospiegel ist. Nach dem Einsatz informieren Sie die Polizei über den Vorfall und klären den Unfall ab.

   ☐ Sie benachrichtigen die Leitstelle über den Vorfall und hängen einen Zettel mit allen wichtigen Informationen an den PKW, da der Halter des PKW nicht vor Ort ist. Sie informieren die Polizei über den Vorfall und fahren weiter zum Einsatzort. Nach dem Einsatz klären Sie mit der Polizei den Unfall ab.

   ☐ Sie informieren die Leitstelle über den Vorfall. Da jedoch keine Person verletzt wurde, fahren Sie zügig weiter zum Einsatzort, da ein Menschenleben wichtiger als ein Autospiegel ist. Nach Beendigung des Einsatzes fahren Sie bei der Polizeistation vorbei und klären den Unfall ab.

   ☐ Sie informieren die Leitstelle über den Vorfall und bitten sie, ein anderes Rettungsmittel zum Einsatzort zu schicken, da Sie erst alles abklären müssen. Da der Halter des PKW nicht vor Ort ist, rufen Sie die Polizei und warten vor Ort auf das Eintreffen der Polizeikollegen.

   ☐ Sie informieren die Leitstelle über den Vorfall. Da keiner verletzt wurde, hängen Sie einen Zettel mit allen wichtigen Informationen zum Unfall an den PKW. Dort ist auch vermerkt, dass es sich um eine eilige Einsatzfahrt handelt und Sie bitten den Fahrzeughalter, sich bei der Polizei zu melden.

6. Das Risiko eines Verkehrsunfalls bei einer Fahrt mit Sondersignalen ist typischerweise höher als bei Normalfahrten. Welche der folgenden Aussagen ist richtig? *[nur eine Antwort]*
   Im Vergleich zu Normalfahrten treten Unfälle bei Sondersignalfahrten:

   ☐ mit Sachschaden 10-fach häufiger auf

   ☐ mit Schwerverletzten 8-fach häufiger auf

   ☐ mit Todesfolge 2-fach häufiger auf

   ☐ mit Schwerverletzten 17-fach häufiger auf

   ☐ mit Todesfolge 7-fach häufiger auf

Additional file 2. Wissenstest-Version A

7. Wer entscheidet darüber, ob Sondersignale eingesetzt werden? *[nur eine Antwort]*

   ☐ Leitstelle

   ☐ Notarzt/Notärztin

   ☐ Rettungsassistent/Rettungsassistentin

   ☐ Einsatzfahrer/Einsatzfahrerin

   ☐ Polizei

8. Wie breit ist das zentrale Blickfeld, in dem Menschen scharf sehen können? *[nur eine Antwort]*

   ☐ ca. 28° ☐ ca. 20° ☐ ca. 15° ☐ ca. 7° ☐ ca. 2°

9. Stellen Sie sich folgende Situation vor: Ein Einsatzfahrer kommt morgens 5:45 Uhr zum Dienst. Zu Beginn überprüfen er und sein Kollege den RTW auf Vollständigkeit. Beide regen sich darüber auf, dass das Vorgängerteam so gut wie nichts nach dem Verbrauch aufgefüllt hat. Der Piepser ruft die beiden noch während des Auffüllens in den Einsatz zu einem Verkehrsunfall mit eingeklemmten Personen. Über Funk informiert die Leitstelle das Team darüber, dass die Feuerwehr unterwegs ist, der Notarzt aber erst in 40 min da sein kann. Im verunfallten Fahrzeug befinden sich zwei Erwachsene und ein Kind. Auf der Anfahrt gerät das Team in den Berufsverkehr und kommt nur schwerlich voran.

   Bitte kreuzen Sie alle Aussagen an, die Ihrer Meinung nach wahrscheinlich stimmen. *[Mehrfachantworten möglich]*

   ☐ Müdigkeit kann die Aufmerksamkeitsleistung verringern. Eine Einsatzfahrt ist jedoch so kurz, dass sie davon nicht beeinträchtigt wird.

   ☐ Der Fahrer ist wegen der Aufregung über die Kollegen genervt und fährt deshalb offensiver.

   ☐ Dass eine junge Familie vom Verkehrsunfall betroffen ist, hat keinen Einfluss auf die Entscheidung, wie vorsichtig zum Einsatzort gefahren wird.

   ☐ Dass die anderen Autofahrer im Berufsverkehr nicht genug Platz machen, stresst und lässt den Fahrer dichter auffahren.

10. Eine Aufprallgeschwindigkeit von 50km/h entspricht etwa welcher Fallhöhe? *[nur eine Antwort]*

    ☐ ca. 2m ☐ ca. 5m ☐ ca. 8m ☐ ca. 10m ☐ ca. 14m

Additional file 2. Wissenstest-Version A

11. Stellen Sie sich folgende Situation vor: Der Einsatzfahrer fährt mit eingeschaltetem Blaulicht zum Notfalleinsatz. 13,5m vor der Kreuzung, die für ihn „rot“ anzeigt, schaltet er das Signalhorn für die Dauer von einer Tonfolge (ca. 3 Sekunden) dazu. Ein querender PKW-Fahrer, für den die Ampel „grün“ zeigt, nimmt das Einsatzfahrzeug zu spät wahr. Es kommt auf der Kreuzung zur Kollision.

    Wer ist Ihrer Ansicht nach in welchem Umfang für die Kollision haftbar? *[nur eine Antwort]*

    ☐ 100% Einsatzfahrer

    ☐ 67% Einsatzfahrer und 33% PKW-Fahrer

    ☐ jeweils 50%

    ☐ 33% Einsatzfahrer und 67% PKW-Fahrer

    ☐ 100% PKW-Fahrer

12. Stellen Sie sich folgende Situation vor: Ein Motorroller-Fahrer fährt in einer Linkskurve mit ca. 40km/h. Er hört über Kopfhörer Musik. Ein Einsatzfahrzeug kommt aus der Gegenrichtung mit eingeschaltetem Blaulicht und Signalhorn straßenmittig mit ca. 35km/h entgegen. Bei Wahrnehmung des Signalhorns orientiert der Roller-Fahrer sich zunächst nach hinten. Erst als er wieder nach vorn schaut, sieht er das Einsatzfahrzeug. Trotz Ausweichversuche beider Fahrzeuge kommt es zur Kollision.

    Wer ist Ihrer Ansicht nach in welchem Umfang für die Kollision haftbar? *[nur eine Antwort]*

    ☐ 100% Einsatzfahrer

    ☐ 67% Einsatzfahrer und 33% Roller-Fahrer

    ☐ jeweils 50%

    ☐ 33% Einsatzfahrer und 67% Roller-Fahrer

    ☐ 100% Roller-Fahrer

13. Sie fahren mit 30 km/h auf einer Straße. Plötzlich taucht ein Hindernis auf, Sie machen eine Vollbremsung und kommen gerade so vor dem Hindernis zum Stehen. Wie hoch wäre die Aufprallgeschwindigkeit am Hindernis, wenn Sie zu Beginn 50km/h gefahren wären? *[nur eine Antwort]*

    ☐ ca. 30km/h ☐ ca. 35km/h ☐ ca. 40km/h ☐ ca. 45km/h ☐ ca. 50km/h

14. Welche Aussage speziell für den Rettungsdienst ist richtig? *[nur eine Antwort]*

- ☐ Laut §11 (besondere Verkehrslagen) der StVO ist es Einsatzfahrern und Einsatzfahrerinnen erlaubt, bestimmte Verkehrsregeln zu missachten, wenn höchste Eile geboten ist, um Menschenleben zu retten oder schwere gesundheitliche Schäden abzuwenden. Die Verkehrssicherheit muss dabei bewahrt bleiben.
- ☐ Laut §35 (Sonderrechte) der StVO sind Einsatzfahrer und Einsatzfahrerinnen von den Vorschriften der StVO befreit, wenn höchste Eile geboten ist, um Menschenleben zu retten oder schwere gesundheitliche Schäden abzuwenden. Die Verkehrssicherheit muss dabei bewahrt bleiben.
- ☐ Laut §38 (Blaues Blinklicht und gelbes Blinklicht) der StVO sind Einsatzfahrer und Einsatzfahrerinnen von den Vorschriften der StVO befreit, wenn höchste Eile geboten ist, um Menschenleben zu retten oder schwere gesundheitliche Schäden abzuwenden. Um das anzuzeigen, können Sie blaues Blinklicht und das Signalhorn einsetzen. Die Verkehrssicherheit muss dabei bewahrt bleiben.
- ☐ Laut den beiden Paragraphen §35 (Sonderrechte) und §38 (Blaues Blinklicht und gelbes Blinklicht) der StVO haben der Einsatzfahrer/die Einsatzfahrerin das Recht, die Vorschriften der StVO zu missachten, um Menschenleben zu retten oder schwere gesundheitliche Schäden abzuwenden. Die Verkehrssicherheit muss dabei bewahrt bleiben.
- ☐ Laut den beiden Paragraphen §46 (Ausnahmegenehmigung und Erlaubnis) und §38 (Blaues Blinklicht und gelbes Blinklicht) der StVO haben der Einsatzfahrer/die Einsatzfahrerin das Recht, die Vorschriften der StVO zu missachten, um Menschenleben zu retten oder schwere gesundheitliche Schäden abzuwenden. Die Verkehrssicherheit muss dabei bewahrt bleiben.

15. Wie viel länger ist der Anhalteweg wenn Sie 70km/h statt 50km/h fahren? *[nur eine Antwort]*

☐ ca. 5m ☐ ca. 10m ☐ ca. 20m ☐ ca. 35m ☐ ca. 50m

16. Je nachdem, wie schnell jemand unterwegs ist, ändern sich das Blickfeld und der Punkt, der deutlich fixiert werden kann (Fixationspunkt). Es entsteht mit höherer Geschwindigkeit ein Tunnelblick, der Fixationspunkt rückt weiter weg und Details im Umfeld werden weniger wahrgenommen. Wie viele Meter entfernt, schätzen Sie, ist der Fixationspunkt bei 50km/h? *[nur eine Antwort]*

☐ ca. 25m ☐ ca. 40m ☐ ca. 55m ☐ ca. 70m ☐ ca. 80m

Additional file 2. Wissenstest-Version A

17. Welche der folgenden Aussagen können zu einem erhöhten Verkehrsrisiko führen? *[Mehrfachantworten möglich]*

- ☐ Ich finde Verkehrssicherheit sehr wichtig.
- ☐ Ich will um jeden Preis Menschenleben retten.
- ☐ Ich fühle mich beim Fahren eines RTW sicher.
- ☐ Ich fahre gern schnell.
- ☐ Ich finde die meisten Geschwindigkeitsbeschränkungen nachvollziehbar.
- ☐ Ich finde es spannend, RTW zu fahren.
- ☐ Ich empfinde Respekt, wenn ich mit Sonder- und Wegerechten fahre.
- ☐ Ich finde, dass das Einsatzstichwort einen Einfluss auf die Fahrweise haben sollte.

18. Wie hoch ist der maximale Zeitvorteil, wenn Sie ungeachtet anderer Verkehrseinschränkungen auf einer Strecke von 12km statt 80km/h 100km/h fahren? *[nur eine Antwort]*

☐ ca. 30sek ☐ ca. 2min ☐ ca. 5min ☐ ca. 8min ☐ ca. 10min

19. Wie hoch ist das Unfallrisiko für einen Fahrer mit 0,6 Promille Blutalkohol im Vergleich zu einem Fahrer mit 0,0 Promille. *[nur eine Antwort]*

☐ 1,5-fach erhöhtes Risiko ☐ 2-fach erhöhtes Risiko ☐ 2,5-fach erhöhtes Risiko ☐ 3-fach erhöhtes Risiko ☐ 3,5-fach erhöhtes Risiko

20. Stellen Sie sich folgende Situation vor: Sie sind als Fahrer auf einem RTW eingeteilt und sind nun auf dem Weg zu dem vierten Notfall dieser Schicht. Bisher sind Sie durchgehend im Einsatz gewesen. Das Stichwort dieses Notfalls lautet „erkrankte Person“, da die Leitstelle zunächst keine genaueren Informationen bekommen konnte. Es ist 17:30 Uhr und Sie befinden sich direkt nach dem Losfahren im Berufsverkehr. Noch haben Sie 9km in der Stadt vor sich, da kein anderes Einsatzmittel in der Nähe ist. Im Laufe der Fahrt gibt die Leitstelle Ihnen weitere Informationen zum Einsatzort.

    Welche konkreten Maßnahmen können Sie während der Fahrt treffen, um sicher am Einsatzort anzukommen? Bitte schreiben Sie die 5 Ihrer Meinung nach wichtigsten Maßnahmen in Stichpunkten auf.

    1. ______________________________
    2. ______________________________
    3. ______________________________
    4. ______________________________
    5. ______________________________
