# Supplementary material for: Traffic safety knowledge gain of ambulance drivers after simulator-based training
Source: BMC Med Educ. 2022 Mar 30;22:216. doi: 10.1186/s12909-022-03279-w (PMC8969364; doi:10.1186/s12909-022-03279-w)
Supplement: Supplementary file 3 — Additional file 3. Wissenstest-Version B. Shows final knowledge test version B in its original German version. [file 12909_2022_3279_MOESM3_ESM.pdf]

Additional file 3. Wissenstest-Version B

1. Bei wie viel Prozent der Unfälle mit Blaulichtbeteiligung sind die Einsatzfahrer/-innen Hauptverursacher? *[nur eine Antwort]*

   ☐ ca. 78% ☐ ca. 65% ☐ ca. 49% ☐ ca. 37% ☐ ca. 30%

2. Auf welcher rechtlichen Basis ist das Verhalten der anderen Verkehrsteilnehmer bei Sondersignal-Fahrten geregelt? *[nur eine Antwort]*

   ☐ Laut §11 (besondere Verkehrslagen) der StVO darf das Blaulicht und Martinshorn nur verwendet werden, wenn höchste Eile geboten ist, um Menschenleben zu retten oder schwere gesundheitliche Schäden abzuwenden. Es signalisiert anderen Verkehrsteilnehmern, dass diese sofort freie Bahn zu schaffen haben.

   ☐ Laut §35 (Sonderrechte) der StVO darf das Blaulicht und Martinshorn nur verwendet werden, wenn höchste Eile geboten ist, um Menschenleben zu retten oder schwere gesundheitliche Schäden abzuwenden. Es signalisiert anderen Verkehrsteilnehmern, dass diese sofort freie Bahn zu schaffen haben.

   ☐ Laut §38 (Blaues Blinklicht und gelbes Blinklicht) der StVO darf das Blaulicht und Martinshorn nur verwendet werden, wenn höchste Eile geboten ist, um Menschenleben zu retten oder schwere gesundheitliche Schäden abzuwenden. Es signalisiert anderen Verkehrsteilnehmern, dass diese sofort freie Bahn zu schaffen haben.

   ☐ Laut den beiden Paragraphen §35 (Sonderrechte) und §38 (Blaues Blinklicht und gelbes Blinklicht) der StVO haben der Einsatzfahrer/die Einsatzfahrerin das Recht, die Vorschriften der StVO zu missachten, um Menschenleben zu retten oder schwere gesundheitliche Schäden abzuwenden. Beide regeln die Nutzung der Sondersignale während der Fahrt und ordnen anderen Verkehrsteilnehmern an, sofort freie Bahn zu schaffen.

   ☐ Laut den beiden Paragraphen §46 (Ausnahmegenehmigung und Erlaubnis) und §38 (Blaues Blinklicht und gelbes Blinklicht) der StVO haben der Einsatzfahrer/die Einsatzfahrerin das Recht, die Vorschriften der StVO zu missachten, um Menschenleben zu retten oder schwere gesundheitliche Schäden abzuwenden. Beide regeln die Nutzung der Sondersignale während der Fahrt und ordnen anderen Verkehrsteilnehmern an, sofort freie Bahn zu schaffen.

3. Wie hoch ist der maximale Zeitvorteil, wenn Sie ungeachtet anderer Verkehrseinschränkungen auf einer Strecke von 6km statt 50km/h 70km/h fahren? *[nur eine Antwort]*

   ☐ ca. 30sek ☐ ca. 2min ☐ ca. 5min ☐ ca. 8min ☐ ca. 10min

4. Eine Aufprallgeschwindigkeit von 50km/h entspricht etwa welcher Fallhöhe? *[nur eine Antwort]*

   ☐ ca. 7m ☐ ca. 10m ☐ ca. 14m ☐ ca. 20m ☐ ca. 27m

Additional file 3. Wissenstest-Version B

5. Das Risiko eines Verkehrsunfalls bei einer Fahrt mit Sondersignalen ist typischerweise höher als bei Normalfahrten. Welche der folgenden Aussagen ist richtig? *[nur eine Antwort]*
   Im Vergleich zu Normalfahrten treten Unfälle bei Sondersignalfahrten:

   ☐ mit Todesfolge 2-fach häufiger auf

   ☐ mit Schwerverletzten 17-fach häufiger auf

   ☐ mit Sachschaden 10-fach häufiger auf

   ☐ mit Schwerverletzten 8-fach häufiger auf

   ☐ mit Todesfolge 7-fach häufiger auf

6. Stellen Sie sich folgende Situation vor: Ein Einsatzfahrer kommt morgens 5:45 Uhr zum Dienst. Zu Beginn überprüfen er und sein Kollege den RTW auf Vollständigkeit. Beide regen sich darüber auf, dass das Vorgängerteam so gut wie nichts nach dem Verbrauch aufgefüllt hat. Der Piepser ruft die beiden noch während des Auffüllens in den Einsatz zu einem Verkehrsunfall mit eingeklemmten Personen. Über Funk informiert die Leitstelle das Team darüber, dass die Feuerwehr unterwegs ist, der Notarzt aber erst in 40 min da sein kann. Im verunfallten Fahrzeug befinden sich zwei Erwachsene und ein Kind. Auf der Anfahrt gerät das Team in den Berufsverkehr und kommt nur schwerlich voran.

   Bitte kreuzen Sie alle Aussagen an, die Ihrer Meinung nach wahrscheinlich stimmen. *[Mehrfachantworten möglich]*

   ☐ Der Fahrer entscheidet schneller zu fahren, um möglichst zeitnah effektive Hilfe leisten zu können, da der Notarzt erst später eintreffen kann.

   ☐ Der Fahrer ist wegen der Unterbrechung in Gedanken noch beim Auffüllen und fährt daher langsamer.

   ☐ Dass die Feuerwehr unterwegs ist, lässt den Fahrer vorsichtiger fahren, da Hilfe frühzeitig bei der Familie sein wird.

   ☐ Dass die anderen Autofahrer im Berufsverkehr nicht genug Platz machen, stresst und lässt den Fahrer dichter auffahren.

Additional file 3. Wissenstest-Version B

7. Stellen Sie sich folgende Situation vor: Ein PKW ordnet sich zum Abbiegen in eine Einfahrt links auf seiner Spur ein und setzt den Blinker. Der Fahrer des PKW konzentriert sich vor dem Abbiegen auf den Gegenverkehr und sieht und hört das von hinten kommende Einsatzfahrzeug mit Blaulicht und Signalhorn nicht. Dieses fährt mit etwa 70 bis 80 km/h und überholt den PKW links, also auf der Gegenspur, in dem Moment wo dieser abbiegt. Es kommt zur Kollision.

   Wer ist Ihrer Ansicht nach in welchem Umfang für die Kollision haftbar? *[nur eine Antwort]*

   - ☐ 100% Einsatzfahrer
   - ☐ 67% Einsatzfahrer und 33% PKW-Fahrer
   - ☐ jeweils 50%
   - ☐ 33% Einsatzfahrer und 67% PKW-Fahrer
   - ☐ 100% PKW-Fahrer

8. Stellen Sie sich folgende Situation vor: Der Einsatzfahrer nähert sich mit Blaulicht und Signalhorn der für ihn „roten“ Ampel einer Kreuzung. Ein von rechts kommender LKW-Fahrer hält auf seiner linken Spur an. Der LKW verdeckt für den Einsatzfahrer die Sicht auf die zweite Spur und den dort fahrenden PKW; für den PKW-Fahrer ist die Sicht auf das Einsatzfahrzeug verdeckt. Aufgrund lauter Musik nimmt der PKW-Fahrer auch das Signalhorn des Einsatzfahrzeuges nicht wahr. Es kommt auf der Kreuzung zur Kollision.

   Wer ist Ihrer Ansicht nach in welchem Umfang für die Kollision haftbar? *[nur eine Antwort]*

   - ☐ 100% Einsatzfahrer
   - ☐ 67% Einsatzfahrer und 33% PKW-Fahrer
   - ☐ jeweils 50%
   - ☐ 33% Einsatzfahrer und 67% PKW-Fahrer
   - ☐ 100% PKW-Fahrer

9. Wie hoch ist das Unfallrisiko für einen Fahrer mit 0,6 Promille Blutalkohol im Vergleich zu einem Fahrer mit 0,0 Promille. *[nur eine Antwort]*

   ☐ 2-fach erhöhtes Risiko ☐ 3-fach erhöhtes Risiko ☐ 4-fach erhöhtes Risiko ☐ 5-fach erhöhtes Risiko ☐ 6-fach erhöhtes Risiko

10. Wie viel länger ist der Anhalteweg wenn Sie 70km/h statt 50km/h fahren? *[nur eine Antwort]*

   ☐ ca.12m ☐ ca. 20m ☐ ca. 33m ☐ ca. 45m ☐ ca. 58m

Additional file 3. Wissenstest-Version B

11. Wie breit ist das zentrale Blickfeld, in dem Menschen scharf sehen können? *[nur eine Antwort]*

☐ ca. 2° ☐ ca. 7° ☐ ca. 15° ☐ ca. 20° ☐ ca. 28°

12. An welchen Orten finden die meisten Unfälle bei Fahrten unter Sonder- und Wegerecht statt? Bitte wählen Sie die korrekte Reihenfolge für absteigende Unfallhäufigkeit aus beginnend mit dem Ort mit der größten Unfallhäufigkeit! *[nur eine Antwort]*

☐ Kreuzung, Kurve, Gerade, Einmündung, Ausfahrt

☐ Gerade, Kreuzung, Ausfahrt, Kurve, Einmündung

☐ Kreuzung, Gerade, Kurve, Einmündung, Ausfahrt

☐ Gerade, Kurve, Kreuzung, Ausfahrt, Einmündung

☐ Kreuzung, Gerade, Einmündung, Ausfahrt, Kurve

13. Welche der folgenden Aussagen können zu einem erhöhten Verkehrsrisiko führen? *[Mehrfachantworten möglich]*

☐ Ich will um jeden Preis Menschenleben retten.

☐ Ich weiß, dass ich durch diesen Einsatz Überstunden machen muss.

☐ Ich fühle mich beim Fahren eines RTW sicher.

☐ Ich versuche immer kraftstoffsparend zu fahren.

☐ Ich fahre gern große Autos mit viel PS.

☐ Ich konzentriere mich voll auf den Verkehr, wenn ich mit Sondersignalen fahre.

☐ Ich verlasse mich darauf, dass andere Fahrzeuge mir Platz machen, wenn ich mit Sondersignal fahre.

☐ Ich finde das Fahrzeughandling eines RTW genauso einfach wie das eines PKW.

Additional file 3. Wissenstest-Version B

14. Stellen Sie sich folgende Situation vor: Sie fahren einen RTW auf dem Weg zum Einsatzort mit dem Stichwort „Atemprobleme bei Kind“. An einer roten Ampel tasten Sie sich in die Kreuzung, alle PKW an der Ampel bleiben stehen. Mitten auf der Kreuzung sehen Sie, dass ein PKW Sie nicht wahrgenommen hat und einem anderen, bereits stehenden PKW auffährt. Sie unterbrechen die Fahrt und verschaffen sich einen Überblick über die Situation. Welche der folgenden Vorgehensweisen ist im Weiteren die beste? *[nur eine Antwort]*

☐ Da keiner schwer verletzt ist, informieren Sie die Polizei über den Vorfall. Sie sorgen dafür, dass die Unfallstelle abgesichert wird. Da Sie am Einsatzort dringend erwartet werden und nicht in den Unfall verwickelt waren, fahren Sie weiter zum Einsatzort, um dem Kind schnellstmöglich zu helfen.

☐ Sie informieren die Leitstelle über den Vorfall und sorgen für die Absicherung der Unfallstelle. Die beteiligten Personen haben nur sehr geringfügige Verletzungen und können die Unfallfahrzeuge selbst an die Seite fahren, weshalb Sie sich entschließen, weiter zum Einsatzort zu fahren.

☐ Da keiner verletzt ist, können die Unfallbeteiligten ihre Fahrzeuge selbst an die Seite fahren. Da Sie vermuten, dass Sie den Unfall mit verursacht haben könnten, bitten Sie die Leitstelle, ein anderes Rettungsmittel zum eigentlichen Einsatzort zu schicken und warten vor Ort auf die Polizei, um alles abzuklären.

☐ Wenn es Verletzte gibt, versorgen Sie die Verletzten. Sie sorgen dafür, dass die Unfallstelle abgesichert wird und entscheiden je nach Schwere der Verletzungen, die Fahrt fortzusetzen. Nach Beendigung des Einsatzes informieren Sie die Polizei über Ihren Einfluss auf den Unfall.

☐ Sie benachrichtigen die Leitstelle und die Polizei. Wenn es Verletzte gibt, versorgen Sie die Verletzten und sichern die Unfallstelle. Mit der Leitstelle klären Sie ab, ob die Fahrt fortgesetzt werden kann. Nach Beendigung des Einsatzes informieren Sie die Polizei über Ihren Einfluss auf den Unfall.

15. Wer entscheidet darüber, ob Sondersignale eingesetzt werden? *[nur eine Antwort]*

☐ Polizei

☐ Einsatzfahrer/Einsatzfahrerin

☐ Leitstelle

☐ Notarzt/Notärztin

☐ Rettungsassistent/Rettungsassistentin

16. Je nachdem, wie schnell jemand unterwegs ist, ändern sich das Blickfeld und der Punkt, der deutlich fixiert werden kann (Fixationspunkt). Es entsteht mit höherer Geschwindigkeit ein Tunnelblick, der Fixationspunkt rückt weiter weg und Details im Umfeld werden weniger wahrgenommen. Wie viele Meter entfernt, schätzen Sie, ist der Fixationspunkt bei 50km/h? *[nur eine Antwort]*

☐ ca. 60m ☐ ca. 40m ☐ ca. 30m ☐ ca. 15m ☐ ca. 3m

Additional file 3. Wissenstest-Version B

17. Ordnen Sie die Quellen für Unfallursachen in die richtige Reihenfolge, indem Sie die entsprechende Zahl von 1-4 davor schreiben (1=häufigste Ursache, 4=seltenste Ursache).

    ______ Verkehrsregelkenntnis

    ______ Wahrnehmung und Gefahrenerkennung

    ______ Fahrzeugbeherrschung

    ______ Fahreinstellung und -motivation

18. Wie müssen Fahrten unter „Sonderrechten“ (§35 der StVO) anderen Verkehrsteilnehmern deutlich gemacht werden? *[nur eine Antwort]*

    ☐ gar nicht

    ☐ durch blaues Blinklicht

    ☐ durch blaues Blinklicht und das Martinshorn

    ☐ durch das Martinshorn

    ☐ durch blaues Blinklicht und Warnblinklicht

19. Sie fahren mit 30 km/h auf einer Straße. Plötzlich taucht ein Hindernis auf, Sie machen eine Vollbremsung und kommen gerade so vor dem Hindernis zum Stehen. Wie hoch wäre die Aufprallgeschwindigkeit am Hindernis, wenn Sie zu Beginn 50km/h gefahren wären? *[nur eine Antwort]*

    ☐ ca. 10km/h ☐ ca. 20km/h ☐ ca. 30km/h ☐ ca. 40km/h ☐ ca. 50km/h

20. Stellen Sie sich folgende Situation vor: Sie sind als Fahrer auf einem RTW eingeteilt und sind nun auf dem Weg zu dem vierten Notfall dieser Schicht. Bisher sind Sie durchgehend im Einsatz gewesen. Das Stichwort dieses Notfalls lautet „erkrankte Person“, da die Leitstelle zunächst keine genaueren Informationen bekommen konnte. Es ist 17:30 Uhr und Sie befinden sich direkt nach dem Losfahren im Berufsverkehr. Noch haben Sie 9km in der Stadt vor sich, da kein anderes Einsatzmittel in der Nähe ist. Im Laufe der Fahrt gibt die Leitstelle Ihnen weitere Informationen zum Einsatzort.

    Welche konkreten Maßnahmen können Sie während der Fahrt treffen, um sicher am Einsatzort anzukommen? Bitte schreiben Sie die 5 Ihrer Meinung nach wichtigsten Maßnahmen in Stichpunkten auf.

    1. ______________________________
    2. ______________________________
    3. ______________________________
    4. ______________________________
    5. ______________________________
