# Supplementary material for: Traffic safety knowledge gain of ambulance drivers after simulator-based training
Source: BMC Med Educ. 2022 Mar 30;22:216. doi: 10.1186/s12909-022-03279-w (PMC8969364; doi:10.1186/s12909-022-03279-w)
Supplement: Supplementary file 4 — Additional file 4. Knowledge test items version A and B – Ad hoc translation (not used or validated). Shows the parallel test items of both final knowledge test version A and B ad hoc translated into English. The English version is not validated nor was it used for study. The correct answers are highlighted in italics with an additional *. [file 12909_2022_3279_MOESM4_ESM.pdf]

Additional file 4. Knowledge test items version A and B – Ad hoc Translation (not used or validated); answers in italics with additional *show correct answer

A1/B12: In which places do most accidents occur when driving under special rights and rights of way? Please select the correct order for descending accident frequency starting with the location with the highest accident frequency! *[only one answer]*

A	B
☐ *crossroads, straight stretch, bend, junction, exit**	☐ crossroads, bend, straight stretch, junction, exit
☐ straight stretch, crossroads, exit, bend, junction	☐ straight stretch, crossroads, exit, bend, junction
☐ crossroads, straight stretch, junction, exit, bend	☐ *crossroads, straight stretch, bend, junction, exit**
☐ crossroads, bend, straight stretch, junction, exit	☐ straight stretch, bend, crossroads, exit, junction
☐ straight stretch, bend, crossroads, exit, junction	☐ crossroads, straight stretch, junction, exit, bend

A2/B17: Put the sources of accident causes in the right order by writing the corresponding number from 1-4 in front of them (1=most frequent cause, 4=rarest cause).

A	B
______ Control of the vehicle	______ Knowledge of traffic regulations
______ Driving attitude and motivation	______ Perception and risk recognition
______ Knowledge of traffic regulations	______ Control of the vehicle
______ Perception and risk recognition	______ Driving attitude and motivation

* Correct answer in both versions: 1=Driving attitude and motivation; 2=Perception and risk recognition; 3=Control of the vehicle; 4=Knowledge of traffic regulations

A3/B18: How must drives under …

A: „rights of way“ (§38 StVO) be signaled to other road users? *[only one answer]*	B: „special rights“ (§35 StVO) be signaled to other road users? *[only one answer]*
☐ *not at all* (*B)<br>☐ only by blue lights<br>☐ *by blue lights and sirens* (*A)<br>☐ only by sirens<br>☐ by blue lights and hazard warning lights	

A4/B1: In what percentage of accidents involving blue light driving are ambulance drivers the main cause? *[only one answer]*

A	B
☐ approx. 30%	☐ approx. 78%
☐ approx. 37%	☐ *approx. 65%**
☐ approx. 49%	☐ approx. 49%
☐ *approx. 65%**	☐ approx. 37%
☐ approx. 78%	☐ approx. 30%

Additional file 4. Knowledge test items version A and B – Ad hoc Translation (not used or validated); answers in italics with additional *show correct answer

A5/B14: Imagine the following situation: You are driving a rescue transport vehicle (RTV) to a scene with the alerting keyword „child with breathing problems"…

A … In a narrow street, a car is parked on the right-hand side a little further into the street than the rest of the vehicles. You accidentally knock off the left mirror of the car. You interrupt the drive and get an overview of the situation. Which of the following is the best way to proceed? *[only one answer]*	B … At a red light, you feel your way into the intersection, all cars at the traffic light stop. In the middle of the intersection, you see that a car has failed to notice you and is rear-ending another car that had already stopped. You interrupt the drive and get an overview of the situation. Which of the following is the best way to proceed? *[only one answer]*
☐ With only the mirror damaged, you decide to continue to the scene, as a human life is more important than a car mirror. After the operation, you inform the police about the incident and clarify the accident.<br>☐ *You notify the dispatch center about the incident and attach a note with all important information to the car, as the owner of the car is not on site. You inform the police about the incident and continue to the scene. After the operation, you clarify the accident with the police*.*<br>☐ You notify the dispatch center about the incident. However, since no person was injured, you continue to the scene quickly, as a human life is more important than a car mirror. After the operation is completed, you drive by the police station and clarify the accident.<br>☐ You notify the dispatch center about the incident and ask them to send another rescue vehicle to the scene, as you need to clarify matters first. Since the owner of the car is not there, you call the police and wait on site for the arrival of police colleagues.<br>☐ You notify the dispatch center about the incident. Since no one was injured, you put a note on the car with all the important information about the accident. It also says that it is an urgent emergency drive and you ask the vehicle owner to contact the police.	☐ As no one is seriously injured, you inform the police about the incident. You make sure that the accident site is secured. As you are awaited urgently at the scene and were not involved in the accident, you drive on to the scene to help the child as quickly as possible.<br>☐ You notify the dispatch center of the incident and make sure that the accident site is secured. The persons involved have only very minor injuries and are able to drive the accident vehicles to the roadside themselves, which is why you decide to continue to the scene.<br>☐ As no one is injured, the parties involved in the accident can pull their vehicles over themselves. As you suspect that you may have contributed to the accident, you ask the dispatch center to send another rescue vehicle to the actual scene and wait for the police on site to clarify everything.<br>☐ If there are injured people, you take care of them. You make sure that the accident site is secured and, depending on the severity of the injuries, decide whether to continue the drive to the scene. After the end of the operation, you inform the police about your contribution to the accident.<br>☐ *You notify the dispatch center and the police. If there are injured people, you take care of them und secure the accident site. You check with the dispatch center whether the journey can be continued. After the end of the operation, you inform the police about your contribution to the accident*.*

A6/B5: The risk of a traffic accident when driving with warning lights and sirens is typically higher than with normal drives. Which of the following statements is correct? *[only one answer]*
Compared to normal drives, during driving with warning lights and sirens accidents ….

A	B
☐ with property damage occur 10 times more frequently<br>☐ *with serious injuries occur 8 times more frequently**<br>☐ with fatalities occur 2 times more frequently<br>☐ with serious injuries occur 17 times more frequently<br>☐ with fatalities occur 7 times more frequently	☐ with fatalities occur 2 times more frequently<br>☐ with serious injuries occur 17 times more frequently<br>☐ with property damage occur 10 times more frequently<br>☐ *with serious injuries occur 8 times more frequently**<br>☐ with fatalities occur 7 times more frequently

Additional file 4. Knowledge test items version A and B – Ad hoc Translation (not used or validated); answers in italics with additional *show correct answer

A7/B15: Who decides whether warning lights and sirens are used? *[only one answer]*

A	B
☐ dispatch center<br>☐ emergency physician<br>☐ paramedic<br>☐ *driver of emergency vehicle**<br>☐ police	☐ police<br>☐ *driver of emergency vehicle**<br>☐ dispatch center<br>☐ emergency physician<br>☐ paramedic

A8/B11: How wide is the focal field of vision in which people can see sharply? *[only one answer]*

A	B
☐ approx. 28<br>☐ approx. 20°<br>☐ approx. 15°<br>☐ approx. 7°<br>☐ *approx. 2°**	☐ *approx. 2°**<br>☐ approx. 7°<br>☐ approx. 15°<br>☐ approx. 20°<br>☐ approx. 28

A9/B6: Imagine the following situation: An ambulance driver comes on duty at 5:45 in the morning. At the beginning, he and his colleague check the RTV for completeness. Both are annoyed that the previous team replenished next to nothing after consumption. The beeper calls them to a traffic accident with trapped persons while they are still filling up. The dispatch center informs the team via radio that the fire fighters are on their way, but that the emergency physician will not be there for another 40 minutes. There are two adults and one child in the crashed vehicle. On the way to the crash site, the team gets caught in rush-hour traffic and makes little headway.

Please check all statements that you think are probably right. *[Multiple answers possible]*

A	B
☐ Sleepiness can reduce attentional performance. However, an emergency drive is so short that it is not affected by it.<br>☐ *The driver is peeved because of the commotion about the colleagues and thus drives more offensively*.*<br>☐ The fact that a young family is hit by the road accident has no influence on the decision on how carefully to drive to the scene of the accident.<br>☐ *The fact that other drivers do not make enough room in rush-hour traffic is stressful and makes the driver tailgate*.*	☐ *The driver decides to drive faster in order to be able to provide effective help as soon as possible, as the emergency physician will only arrive later*.*<br>☐ Because of the interruption, the driver is still thinking about filling up and therefore drives more slowly.<br>☐ The fact that the fire fighters are on the way prompts the driver to drive more cautiously, as help will be with the family soon.<br>☐ *The fact that other drivers do not make enough room in rush-hour traffic is stressful and makes the driver tailgate*.*

A10/B4: To what height of fall does an impact speed of 50km/h approximately correspond? *[only one answer]*

A	B
☐ approx. 2m<br>☐ approx. 5m<br>☐ approx. 8m<br>☐ *approx. 10m**<br>☐ approx. 14m	☐ approx. 7m<br>☐ *approx. 10m**<br>☐ approx. 14m<br>☐ approx. 20m<br>☐ approx. 27m

Additional file 4. Knowledge test items version A and B – Ad hoc Translation (not used or validated); answers in italics with additional *show correct answer

A11/B8: Imagine the following situation:

A: The ambulance driver drives to the emergency with his blue lights on. 13.5m before the crossroads, which indicates "red" for him, he switches on the sirens for the duration of one tone sequence (approx. 3 seconds). A crossing car driver, for whom the traffic light shows "green", notices the emergency vehicle too late. They collide at the intersection.	B: The ambulance driver approaches traffic lights at a crossroads, which indicate "red" for him, with warning lights and sirens. A truck driver approaching from the right stops in his left lane. The truck obscures the ambulance driver's view of the second lane and the car driving there; the car driver's view of the ambulance is blocked. Due to loud music, the car driver also fails to notice the sirens of the ambulance. They collide at the intersection.
In your opinion, who is liable for the collision and to what extent? *[only one answer]*<br>☐ *100% ambulance driver* (*A)<br>☐ *67% ambulance driver and 33% car driver* (*B)<br>☐ each 50%<br>☐ 33% ambulance driver und 67% car driver<br>☐ 100% car driver	

A12/B7: Imagine the following situation:

A: A motor scooter rider takes a left-hand bend at approx. 40km/h. He listens to music on his headphones. An emergency vehicle is approaching from the opposite direction with warning lights and sirens on in the middle of the road at approx. 35km/h. When he perceives the sirens, the scooter rider first orients himself to the rear. Only when he looks ahead again does he see the emergency vehicle. Despite attempts by both vehicles to take evasive action, a collision occurs.	B: A car moves left on its lane to turn into a driveway and sets the blinker. The driver of the car concentrates on the oncoming traffic before turning and does not see or hear the emergency vehicle with warning lights and sirens coming from behind. The ambulance is travelling at about 70 to 80 km/h and overtakes the car on the left, i.e. on the opposite lane, at the moment when the car turns. They collide.
In your opinion, who is liable for the collision and to what extent? *[only one answer]*<br>☐ 100% ambulance driver<br>☐ 67% ambulance driver and 33% car driver<br>☐ *each 50%* (*A)<br>☐ *33% ambulance driver und 67% car driver* (*B)<br>☐ 100% car driver	

A13/B19: You are driving at 30 km/h on a road. Suddenly an obstacle appears, you slam on the brakes and come to a halt just in front of the obstacle. What would be the impact speed at the obstacle if you had been driving 50km/h at the start? *[only one answer]*

A	B
☐ approx. 30km/h<br>☐ approx. 35km/h<br>☐ approx. 40km/h<br>☐ approx. 45km/h<br>☐ *approx. 50km/h**	☐ approx. 10km/h<br>☐ approx. 20km/h<br>☐ approx. 30km/h<br>☐ approx. 40km/h<br>☐ *approx. 50km/h**

Additional file 4. Knowledge test items version A and B – Ad hoc Translation (not used or validated); answers in italics with additional *show correct answer

A14/B2:

A: Which statement specifically for the rescue service is correct? *[only one answer]*	B: What is the legal basis for regulating the behavior of other road users when driving with warning lights and sirens? *[only one answer]*
☐ According to §11 (special traffic situations) of the Road Traffic Regulations (StVO) ambulance drivers are allowed to disregard certain traffic rules if utmost urgency is required in order to save human lives or avert serious damage to health. Road safety must be preserved in the course of this.<br>☐ *According to §35 (special rights) of the StVO, ambulance drivers are exempt from the regulations of the StVO if utmost urgency is required to save human lives or avert serious damage to health. Road safety must be preserved in the course of this*.*<br>☐ According to §38 (blue flashing light and yellow flashing light) of the StVO, ambulance drivers are exempt from the regulations of the StVO if utmost urgency is required to save human lives or avert serious damage to health. To indicate this, they are allowed to use blue lights and siren. Road safety must be preserved in the course of this.<br>☐ According to the two paragraphs §35 (special rights) and §38 (blue flashing light and yellow flashing light) of the StVO, the ambulance driver has the right to disregard the regulations of the StVO in order to save human lives or avert serious damage to health. Road safety must be preserved in the course of this.<br>☐ According to the two paragraphs §46 (exemption and permission) and §38 (blue flashing light and yellow flashing light) of the StVO, the ambulance driver has the right to disregard the regulations of the StVO in order to save human lives or avert serious damage to health. Road safety must be preserved in the course of this.	☐ According to §11 (special traffic situations) of the Road Traffic Regulations (StVO), the blue light and siren may only be used if utmost urgency is required to save human lives or avert serious damage to health. It signals to other road users that they must clear the way immediately.<br>☐ According to §35 (special rights) of the StVO, the blue light and siren may only be used if utmost urgency is required to save human lives or avert serious damage to health. It signals to other road users that they must clear the way immediately.<br>☐ *According to §38 (blue flashing light and yellow flashing light) of the StVO, the blue light and siren may only be used if utmost urgency is required to save human lives or avert serious damage to health. It signals to other road users that they must clear the way immediately*.*<br>☐ According to the two paragraphs §35 (special rights) and §38 (blue flashing light and yellow flashing light) of the StVO, the emergency driver has the right to disregard the regulations of the StVO in order to save human lives or avert serious damage to health. Both regulate the use of the warning lights and sirens while driving and order other road users to clear the way immediately.<br>☐ According to the two paragraphs §46 (exemption and permission) and §38 (blue flashing light and yellow flashing light) of the StVO, the emergency driver has the right to disregard the regulations of the StVO in order to save human lives or avert serious damage to health. Both regulate the use of the warning lights and sirens while driving and order other road users to clear the way immediately.

A15/B10: How much longer is the stopping distance if you drive 70km/h instead of 50km/h? *[only one answer]*

A	B
☐ approx. 5m<br>☐ approx. 10m<br>☐ *approx. 20m**<br>☐ approx. 35m<br>☐ approx. 50m	☐ approx. 12m<br>☐ *approx. 20m**<br>☐ approx. 33m<br>☐ approx. 45m<br>☐ approx. 58m

Additional file 4. Knowledge test items version A and B – Ad hoc Translation (not used or validated); answers in italics with additional *show correct answer

A16/B16: Depending on how fast someone is moving, the field of vision and the point that can be clearly focused on (fixation point) change. Tunnel vision develops at a higher speed, the fixation point moves further away and details in the surroundings are perceived to a lesser extent. How many meters away do you estimate the fixation point is at 50km/h? _[only one answer]_

A	B
☐ approx. 25m<br>☐ _approx. 40m*_<br>☐ approx. 55m<br>☐ approx. 70m<br>☐ approx. 80m	☐ approx. 60m<br>☐ _approx. 40m*_<br>☐ approx. 30m<br>☐ approx. 15m<br>☐ approx. 3m

A17/B13: Which of the following statements can lead to an increased traffic risk? [_Multiple answers possible]_

A	B
☐ I think road safety is very important.<br>☐ _I want to save lives at all costs.*_<br>☐ _I feel safe driving an RTV_.*<br>☐ _I like driving fast_.*<br>☐ I think most of the speed limits are reasonable.<br>☐ _I find it exciting to drive RTV_.*<br>☐ I have respect for rides with special rights and rights of way.<br>☐ _I think that the alerting keyword should have an influence on the driving style_.*	☐ _I want to save lives at all costs_.*<br>☐ I know that I have to work overtime because of this mission.<br>☐ _I feel safe driving an RTV_.*<br>☐ I always try to drive in a fuel-efficient way.<br>☐ _I like driving big cars with lots of horsepower_.*<br>☐ I concentrate fully on the traffic when I drive with warning lights and sirens.<br>☐ _I trust other vehicles to make way for me when I am driving with warning lights and sirens_.*<br>☐ _I think the vehicle handling of an RTV is just as easy as that of a car_.*

A18/B3:

A: What is the maximum time advantage if you drive 100km/h instead of 80km/h on a stretch of 12km, regardless of other traffic restrictions? _[only one answer]_	B: What is the maximum time advantage if you drive 70km/h instead of 50km/h on a stretch of 6km, regardless of other traffic restrictions? _[only one answer]_
☐ approx. 30sec<br>☐ _approx. 2min*_<br>☐ approx. 5min<br>☐ approx. 8min<br>☐ approx. 10min	

A19/B9: What is the accident risk for a driver with 0.6 per mille blood alcohol compared to a driver with 0.0 per mille? _[only one answer]_

A	B
☐ 1.5-fold increased risk<br>☐ 2-fold increased risk<br>☐ 2.5-fold increased risk<br>☐ _3-fold increased risk*_<br>☐ 3.5-fold increased risk	☐ 2-fold increased risk<br>☐ _3-fold increased risk*_<br>☐ 4-fold increased risk<br>☐ 5-fold increased risk<br>☐ 6-fold increased risk

Additional file 4. Knowledge test items version A and B – Ad hoc Translation (not used or validated); answers in italics with additional *show correct answer

A20/B20: Imagine the following situation: You are assigned as a driver on an RTV and are now on your way to the fourth emergency of this shift. So far, you have been on duty continuously. The keyword of this emergency is "sick person", as the dispatch center was initially unable to obtain more precise information. It's 5:30 p.m. and you are in rush hour traffic right after you start driving. You still have 9km to drive in the city, as there is no other emergency vehicle in the vicinity. In the course of the drive, the dispatch center gives you further information on the location of the operation.

What concrete measures can you take during the ride to arrive safely at the scene? Please write down in bullet points the five measures you consider most important.

1. ____
2. ____
3. ____
4. ____
5. ____
