# Supplementary material for: Traffic safety knowledge gain of ambulance drivers after simulator-based training
Source: BMC Med Educ. 2022 Mar 30;22:216. doi: 10.1186/s12909-022-03279-w (PMC8969364; doi:10.1186/s12909-022-03279-w)
Supplement: Supplementary file 5 — Additional file 5. Categories of free responses. Shows all categories for free responses of the knowledge test with explanation and examples for correct and incorrect answers. [file 12909_2022_3279_MOESM5_ESM.pdf]

Additional file 5.

Categories of free responses

Categories	Positive/correct sample items	General, non-scored sample items
1. minimize distraction or setting priorities		
During driving it is important to concentrate on driving and the road and not be distracted by other tasks	- delegation to co-driver (navigation, operation)<br>- turning off other disturbing factors (radio, cell phone)	- stay calm<br>- contact control room<br>- discuss information with co-driver
2. route planning		
Using of a useful route to reach operation site.	- think about route before starting<br>- usage of special lanes (bus or taxi lane)<br>- knowing road blockings<br>- consider traffic (rush hour)	
3. usage of right of way and warning light and sirens		
Decisions concerning driving with warning lights and sirens, how to use them and what to consider concerning other road users.	- driving with warning lights AND sirens the whole way<br>- being aware that others still might react unpredictable or late<br>- do not rely on good going situations but still be careful (stopping at red signs)	- be careful with others<br>- be attentive<br>- "special rights" alone without any further elaboration
4. speed and driving physics		
Speed has the highest influence on stopping distances and impact speed. Reducing and adaption of speed is therefore essential.	- do not drive too fast<br>- reduce speed<br>- adapt speed to weather, traffic and driving situations<br>- you have to be able to stop within the visible driving way<br>- drive defensively	- drive anticipatory<br>- stay calm<br>- safety before speed<br>- consider traffic

Categories	Positive sample items	General, non-scored sample items
5. scope of action for other road users<br>Other road users might need more time to orient themselves how to give free way to ambulances. Ambulance drivers have to give other road users enough time to react and show them where they want to drive to make it easier for others to react appropriate.	- early signaling of desired way to drive<br>- drive straight (without changing too much between lanes or possibilities)<br>- waiting if there is no way to drive through<br>- no risky overtaking maneuvers	- respect other road users<br>- be attentive
6. decision behavior<br>The impact of emotional and motivational factors on decision behavior in traffic should be known.	- do not let operation indication affect driving<br>- try to estimate the real risk of situations without assessing the operation<br>- be aware of longer reaction times due to operation stress	- consider traffic<br>- always expect everything<br>- not be provoked by others
7. information processing and personal requirements<br>The amount of information someone is possible to process is dependent of stress. Operation information could negatively influence information processing.	- change driver when he/she is tired<br>- co-driver should additionally warn of dangers<br>- take time to have an overview of the road<br>- accept delays and mistakes of other drivers	- drive relaxed<br>- do not be stressed<br>- stay calm
8. general activities (independent of blue light driving)<br>Actions of passive safety that can be used independently of driving with warning lights and sirens; technical or safety concerning actions of vehicle	- using seat belt<br>- adjust mirrors and seat<br>- drive with lights on<br>- know your vehicle<br>- regular technical inspection of vehicle (lights, breaks, liquids)	- stay calm<br>- always expect everything<br>- using a relaxing seat position
